# Decoding the Heterogeneity of Malignant Gliomas by PET and MRI for Spatial Habitat Analysis of Hypoxia, Perfusion, and Diffusion Imaging: A Preliminary Study

**DOI:** 10.3389/fnins.2022.885291

**Published:** 2022-07-13

**Authors:** Michele Bailo, Nicolò Pecco, Marcella Callea, Paola Scifo, Filippo Gagliardi, Luca Presotto, Valentino Bettinardi, Federico Fallanca, Paola Mapelli, Luigi Gianolli, Claudio Doglioni, Nicoletta Anzalone, Maria Picchio, Pietro Mortini, Andrea Falini, Antonella Castellano

**Affiliations:** ^1^Vita-Salute San Raffaele University, Milan, Italy; ^2^Department of Neurosurgery and Gamma Knife Radiosurgery, IRCCS Ospedale San Raffaele, Milan, Italy; ^3^Neuroradiology Unit and CERMAC, IRCCS Ospedale San Raffaele, Milan, Italy; ^4^Pathology Unit, IRCCS Ospedale San Raffaele, Milan, Italy; ^5^Department of Nuclear Medicine, IRCCS Ospedale San Raffaele, Milan, Italy

**Keywords:** high-grade glioma, PET, MRI, habitats, hypoxia imaging, perfusion-weighted imaging, diffusion-weighted imaging, tumor heterogeneity

## Abstract

**Background:**

Tumor heterogeneity poses major clinical challenges in high-grade gliomas (HGGs). Quantitative radiomic analysis with spatial tumor habitat clustering represents an innovative, non-invasive approach to represent and quantify tumor microenvironment heterogeneity. To date, habitat imaging has been applied mainly on conventional magnetic resonance imaging (MRI), although virtually extendible to any imaging modality, including advanced MRI techniques such as perfusion and diffusion MRI as well as positron emission tomography (PET) imaging.

**Objectives:**

This study aims to evaluate an innovative PET and MRI approach for assessing hypoxia, perfusion, and tissue diffusion in HGGs and derive a combined map for clustering of intra-tumor heterogeneity.

**Materials and Methods:**

Seventeen patients harboring HGGs underwent a pre-operative acquisition of MR perfusion (PWI), Diffusion (dMRI) and ^18^F-labeled fluoroazomycinarabinoside (^18^F-FAZA) PET imaging to evaluate tumor vascularization, cellularity, and hypoxia, respectively. Tumor volumes were segmented on fluid-attenuated inversion recovery (FLAIR) and T1 post-contrast images, and voxel-wise clustering of each quantitative imaging map identified eight combined PET and physiologic MRI habitats. Habitats’ spatial distribution, quantitative features and histopathological characteristics were analyzed.

**Results:**

A highly reproducible distribution pattern of the clusters was observed among different cases, particularly with respect to morphological landmarks as the necrotic core, contrast-enhancing vital tumor, and peritumoral infiltration and edema, providing valuable supplementary information to conventional imaging. A preliminary analysis, performed on stereotactic bioptic samples where exact intracranial coordinates were available, identified a reliable correlation between the expected microenvironment of the different spatial habitats and the actual histopathological features. A trend toward a higher representation of the most aggressive clusters in WHO (World Health Organization) grade IV compared to WHO III was observed.

**Conclusion:**

Preliminary findings demonstrated high reproducibility of the PET and MRI hypoxia, perfusion, and tissue diffusion spatial habitat maps and correlation with disease-specific histopathological features.

## Introduction

Tumor heterogeneity poses a major clinical challenge in brain tumors and its enhanced assessment can be pivotal to understand treatment failure in high-grade gliomas (HGGs) (O’Connor et al., 2015; Stadlbauer et al., 2018b). Indeed, in HGGs, the regional diversity both at the level of DNA mutation and gene expression profiles may ultimately alter phenotype by influencing tumor cell metabolism, apoptosis, and angiogenesis processes (Louis et al., 2016; Komori et al., 2018). At the individual patient level, the heterogeneous biology of malignant gliomas challenges tools that rely on single biopsy criteria for making disease-wide assessments and poses a problem in planning effective subsequent treatments. Furthermore, tumor heterogeneity is reflected in differential and dynamical response during therapy, eventually causing the failure of previously effective alkylating drugs or targeted therapies, as resistant clones invariably emerge and proliferate (Sottoriva et al., 2013; Stadlbauer et al., 2018b).

Non-invasive imaging can provide multiple biomarkers with insight into the malignancy and biology of gliomas. Conventional magnetic resonance imaging (cMRI) is routinely employed in the diagnosis and clinical management of malignant gliomas: beside the tumor features described by cMRI sequences, advanced physiological MRI techniques such as diffusion MRI (dMRI) and perfusion-weighted imaging (PWI) add important structural, physiological and hemodynamic information to measure biological properties quantitatively and non-invasively and correlate with patients’ outcome (Castellano and Falini, 2016). Similarly, positron emission tomography (PET) imaging reflects fundamental metabolic patterns throughout the tumor. In particular, PET using tracers from nitroimidazoles family, such as ^18^F-labeled fluoroazomycinarabinoside (^18^F-FAZA) or ^18^F-labeled fluoromisonidazole (^18^F-FMISO), identifies areas of hypoxic tissue that have been shown as related to treatment resistance, thus negatively impacting on patient outcome and survival (Preibisch et al., 2017; Abdo et al., 2019).

Furthermore, with the application of advanced mathematical modeling, it is also possible to divide neoplasms into definite subregions comprehending clusters of voxels with comparable radiomics features. This method is defined as “habitat imaging” and is considered a way to characterize and measure the heterogeneity of the tumoral microenvironment (Gatenby et al., 2013). Habitat imaging has been currently mainly employed on cMRI sequences, although it could hypothetically be applied to any neuroimaging method, comprising advanced MRI techniques such as PWI and dMRI as well as PET imaging with different radiotracers (Zhou et al., 2014; Hu et al., 2015; Lee et al., 2015a,b; O’Connor et al., 2015; Cui et al., 2016; Sauwen et al., 2016; Dextraze et al., 2017; Kim and Gatenby, 2017; Zhou et al., 2017; Fathi Kazerooni et al., 2018; Fuster-Garcia et al., 2018; Ismail et al., 2018; Juan-Albarracin et al., 2018; Li et al., 2018a,b; John et al., 2019; Juan-Albarracin et al., 2019; Li et al., 2019; Stringfield et al., 2019; Wang et al., 2019; Wei et al., 2019; Zhang et al., 2019; Beig et al., 2020; Del Mar Alvarez-Torres et al., 2020; Verma et al., 2020; Wu et al., 2020; Kim et al., 2021; Park et al., 2021).

To date, the spatial relationship between areas of hypoxia, neoangiogenesis, and altered tissue microstructure and cellularity has not been completely elucidated in HGGs using advanced neuroimaging. The aim of the present study is to evaluate an innovative, PET and MRI approach for the assessment of hypoxia, perfusion, and tissue diffusion in HGG and derive a combined spatial habitat map for clustering of intra-tumor heterogeneity. To the best of our knowledge, this represents the first study combining the aforementioned parameters to define brain tumor habitats.

## Materials and Methods

### Patients

In this study the PET and MRI datasets of twenty patients harboring brain lesions with MRI features suggestive for HGG were analyzed, previously recruited in a prospective study on hypoxia tracers at San Raffaele Scientific Institute from April 2016 to October 2017. The local research ethics committee approved the study, and informed consent was obtained from all patients. All the subjects underwent advanced MRI acquisition and ^18^F-FAZA PET/CT before surgery, except for two patients that were withdrawn from the study due to technical problems related to radiotracer synthesis. Another patient was eventually excluded from the analysis since the final histopathological diagnosis was brain metastases from lung cancer.

### PET/CT

The procedure of ^18^F-FAZA radiotracer production followed the technique previously reported by Savi et al. (2017). Every recruited patient had an intravenous administration of about 372 ± 17 (minimum 340, maximum 407) MBq of ^18^F-FAZA. The PET/CT scan acquisition and analysis were conducted as previously reported by Mapelli and Picchio (2020) and Mapelli et al. (2021).

### MRI Acquisition

Pre-operative MRI datasets were acquired (1–5 days prior to the surgical procedure) on a 1.5 T scanner (Philips Achieva–Philips Healthcare, Best, Netherlands) in 12/17 cases, and on a 3.0 T (Ingenia CX scanner, Philips Healthcare, Best, Netherlands) on 5/17 patients.

**FIGURE 1:**
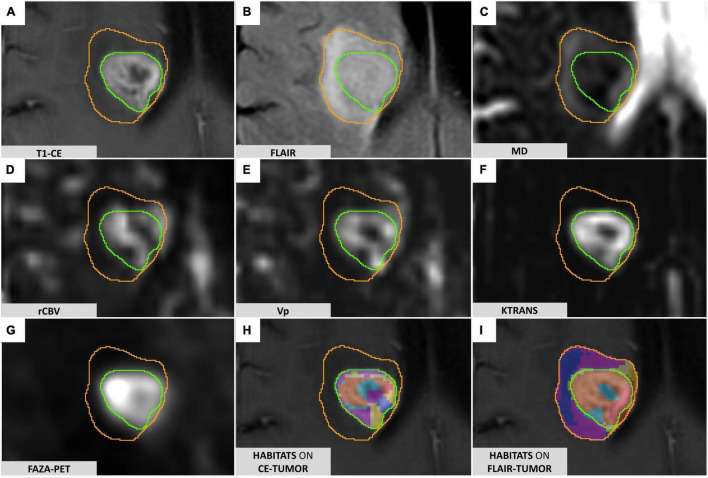
FLAIR-tumor and CE-tumor segmentations on different MRI sequences. **(A)** T1-weighted contrast-enhanced imaging: the CE-tumor volume is contoured in green, including the enhancing tissue and necrotic core only. **(B)** FLAIR images: FLAIR-tumor is contoured in orange, including both the CE-tumor and the peritumoral hyperintensity. **(C)** MD map elaborated from DTI acquisition. **(D)** rCBV map elaborated from DSC MR perfusion acquisition. **(E)** VP map elaborated from DCE MR perfusion acquisition **(F)** Ktrans map elaborated from DCE MR perfusion acquisition. **(G)** 18F-FAZA-PET uptake showing hypoxic areas. **(H)** Prototype of clusters resulting from the application of the spatial habitat imaging algorithm only to the contrast-enhanced tumor volume (CE-tumor); clusters obtained this way were considered too small and fragmented in order to be reliably correlated to histology, and therefore not suitable for the purpose of the present study. **(I)** Habitats obtained applying the spatial habitat imaging algorithm to the FLAIR-tumor volume (which included the contrast-enhanced tumor core along with the surrounding area of FLAIR hyperintensity) were able to divide the whole tumor in a more reproducible way, with larger clusters amenable to a reliable histological correlation with the bioptic samples obtained; for the aforementioned reasons we selected FLAIR-tumor as the final tumor mask to be investigated and all the results presented in our paper are based on its analysis.

Imaging acquisition included the following sequences:

1. 3D-FLAIR (fluid-attenuated inversion recovery) and Contrast-enhanced (CE) 3D-T1 weighted images, for morphological evaluation and tumor segmentation (Figures 1A,B);

2. Diffusion Tensor Imaging (DTI) sequence to detect microstructural features of the tumor tissue. DTI data were obtained using a single-shot echo planar sequence with diffusion gradients applied along 15 or 32 axes, using *b*-values of 0 and 1000 s/mm^2^. After correcting for distortions due to eddy currents and head motion, maps of Mean Diffusivity (MD) (Figure 1C) were derived from DTI acquisitions by fitting the diffusion tensor model within each voxel using the FMRIB Diffusion Toolbox (FDT tool, FMRIB Software Library [FSL] version 6.0.0^1^);
3. Perfusion-weighted magnetic resonance imaging including dynamic contrast-enhanced (DCE) and dynamic susceptibility contrast-enhanced (DSC) MRI, to characterize tumor vascularization and neoangiogenic processes, according to a previously descripted protocol (Santarosa et al., 2016; Anzalone et al., 2018). PWI analysis was performed with Olea Sphere (v 3.0, Olea Medical Solutions, France) to obtain the parametric maps of volume transfer constant (Ktrans) and plasma volume (Vp), derived from DCE, and relative cerebral blood volume (rCBV), derived by DSC, as described in Conte et al. (2017) (Figures 1D–F). Pre-processing steps included automatic motion correction by a rigid-body registration, automatic spatial smoothing, and background segmentation.

The complete acquisition parameters of all the sequences are reported in the Supplementary Tables 1, 2.

### Image Processing

For each patient, all the radiological studies and maps were imported, coregistered to the reference 3D-T1 CE sequence (two consecutive automatic rigid registration processes, with 6 degrees of freedom and 0.1 as percentage of samples, were performed after initial manual adjustment, then carefully checked for the eventual necessity of minor manual correction) and reformatted to the same matrix (voxel 1 × 1 × 1 mm) in the image processing software 3D Slicer^2^ (Fedorov et al., 2012), creating a single dataset for each patient. Two different tumor masks were segmented manually using 3D Slicer, being carefully checked repeatedly by the first author (MB) and an experienced neuroradiologist (AC): one including only the enhancing tissue and necrotic core on the post-gadolinium 3D-T1 images (CE-tumor, green contour in Figure 1A), the second including, additionally, the peritumoral infiltrative edema as identified on 3D-FLAIR images (FLAIR-tumor, orange contour in Figure 1B). Care was taken, while comparing data from multiple coregistered sequences (e.g., Susceptibility-weighted imaging, T2-weighted, PWI maps, MD maps, T1-weighted images with and without contrast enhancement, CT) not to include non-tumoral structures that could jeopardize subsequent analyses (e.g., subarachnoid cisterns, sulci, ventricular space, choroid plexus, ependyma, significant vascular structures, dural folds, calcifications).

The feasibility of deriving a combined spatial habitat map was then exploited by using the automatic Otsu thresholding algorithm method developed in Matlab2019a (The MathWorks, Inc., Natick, MA, United States). The Otsu algorithm was chosen for its simplistic approach in dividing ROI tumoral voxels into two clusters with high and low-intensity (Zhou et al., 2014, 2017; Napel et al., 2018; Stringfield et al., 2019), thus maintaining the clinical significance of each map subregion. The Otsu algorithm calculates a global threshold T from each of the grayscale tumoral ROI (perfusion, diffusion, and hypoxia, Figures 1C–G) by minimizing intra-class intensity variance, or equivalently, by maximizing inter-class variance as described in Otsu (1979).

For each subject the three tumoral ROIs were imported in Matlab2019a and voxels included inside the tumor ROI [excluding those outside the range of mean ± 3 standard deviations, as described in Collewet et al. (2004)] were clustered into two disjoint subregions with the automatic Otsu’s algorithm. Therefore, given three tumor-masked maps (D_1_, D_2_, and D_3_), they were clustered as follows:


D→1[D,1⁢LD]1⁢H,D→2[D,2⁢LD]2⁢H,andD→3[D,3⁢LD]3⁢H

D∩1⁢LD=1⁢H∅,D∩2⁢LD=2⁢H∅,andD∩3⁢LD=3⁢H∅

In this way, 2 clusters for each map (“high” and “low” expression of a parameter) were obtained. Then, spatial habitat maps were composed in each patient by defining as a different ‘habitat’ each possible combination of clusters (see Supplementary Figure 1). Thus, the cluster that groups together all high-intensity cluster sequences (HHH) is defined as:


Habitat(HHH):[D∩1⁢HD∩2⁢HD]3⁢H

The different habitats were eventually represented with different colors (Figures 1H,I) to ease visual identification, yield regions with different combinations of perfusion, diffusion, and hypoxia, supposedly reflecting different physiological microenvironments within the tumor.

### Surgical Procedure and Histopathological Evaluation

All patients underwent the surgical procedure at the Neurosurgery Department of the IRCCS San Raffaele Hospital (Milan, Italy). Patients were considered for maximum safe resection or stereotactic needle biopsy according to tumor location/extension and patient characteristics (e.g., age, comorbidities, and performance status).

In the case of stereotactic biopsy, the procedure commenced with the fixation of the MRI-compatible Leksell stereotactic frame to the patient’s skull (Model G, Elekta, Stockholm, Sweden), under mild intravenous sedation (midazolam) and local anesthesia (lidocaine and mepivacaine). The patient then underwent the acquisition of an axial 3D-T1 MRI after gadolinium administration (Voxel 1 × 1 mm, slice thickness 1 mm, Matrix 256 × 256 mm) using the appropriate fiducial system on a Philips Achieva 1.5 Tesla MR scanner (Philips Medical Systems, Best, Netherlands). In most of the cases, a 3D T2-weighted series was also obtained. All the relevant imaging and parameter maps were then imported into the stereotactic planning software Leksell SurgiPlan^®^ (Elekta Instruments AB, Stockholm, Sweden) and coregistered to the newly acquired stereotactic images.

Since the final tumor habitats were not available to the neurosurgeon pre-operatively, the designation of target sites for biopsy sites and the needle path were planned according to the best clinical practice in order to maximize patient safety while ensuring final diagnosis. During the procedure, a Sedan needle (2.5 mm diameter and 10 mm of bioptic window) was used to acquire a minimum of two bioptic samples. Considering the macroscopical aspect and size of the sample obtained and the procedure-related risks/complications, a median of 3 cylindrical tissue biopsies (median length 8 mm, range 4–11 mm) were obtained. The accuracy of the bioptic sampling was eventually verified in all cases through the co-registration of the pre-operative imaging and planned trajectory with the post-operative CT images inside the SurgiPlan software; the stereotactic coordinates of the exact, final sites of the biopsy were then imported in the 3D Slicer Dataset.

The tissue samples obtained were directly fixed in a 10% formalin solution and referred to the pathology department, where they were processed the same day or the following one if the procedures were performed in the late afternoon. The final diagnosis was established according to the 2016 World Health Organization (WHO) classification (Louis et al., 2016). A descriptive analysis of the microscopic morphology of each sample was performed by an expert neuropathologist (MC) attended by a neurosurgeon (MB), repeating the whole evaluation at three distant time points (blinded from previously reported values and patient’s case) in order to enhance the reproducibility of the results. Additionally, to address the issue of the length variability among different bioptic specimens and to maximize the correspondence with the target point set on the stereotactic planning system, only the central portion (extending 5 mm along the greatest axis of the specimen in its largest section available) was analyzed in each sample.

The following parameters were semi-quantitatively graded similarly to a previously reported work (Mikkelsen et al., 2018), despite the adoption of a higher number of classes dividing each feature in the present analysis: cellularity, the extent of necrosis, and the number of hyperplastic vessels counted. Cellularity was graded with a score from 1 (seemingly normal representation) to 5 (extremely high cellular density). Classes adopted for necrosis (expressed as a rate over the whole area considered for each specimen) were the following: 0 (0%), 1 (≤10%), 2 (11–25%), 3 (26–50%), 4 (51–75%), and 5 (76–100%).

In the 10/17 patients who underwent needle biopsy, the stereotactic coordinates of the sampling sites were imported in the 3D Slicer Dataset as a 5 mm diameter spherical volume, similarly to what was previously described by other authors (Jun et al., 2006; Barajas R. F. et al., 2012; Gates et al., 2019; Rotkopf et al., 2020), in order to take into account the maximum reported accuracy error in Euclidean distance reported in the literature (Roth et al., 2018). Each histopathological specimen, labeled according to the order of resection, was then analyzed in comparison to the corresponding voxels and habitats in the 3D Slicer Dataset.

### Statistical Analysis

Statistical analysis was performed with IBM SPSS Statistics, version 23.0 (IBM Corp., Armonk, NY, United States), Prism 9 version 3.1 (GraphPad Software, LLC) and Matlab2019a (MathWorks, Natick, MA, United States). The threshold of statistical significance was defined as two-sided *P* = 0.05. Distribution normality was assessed with the Kolmogorov–Smirnov test.

The following variables were tested for possible associations: quantitative values across different habitats for each parametric map, CE-tumor volume, FLAIR-tumor volume, representation of the different habitats (absolute volume, rate over total), patient’s age, WHO tumor grading, MGMT methylation, 1p19q codeletion, IDH-1 status. The distribution of mean values of Vp, ^18^F-FAZA and MD parametric maps in each habitat was displayed by means of boxplots.

A Kruskal–Wallis test was conducted to determine if there were differences among different habitats in voxel-wise parametric values for each PET and MRI map. The percentage of habitats’ representation according to tumor grade and molecular status was assessed by boxplots’ visual inspection. Pairwise comparisons were performed using Dunn’s procedure with Bonferroni correction for multiple comparisons.

## Results

### Patients and Lesion Characteristics

The study population (data summarized in Table 1) was composed of 5 female and 12 male patients, with ages ranging from 41 to 81 years old (mean 66 years).

**TABLE 1:** Patient demographic, histopathologic, and survival data.

No.	Age (yrs)	Sex	Tumor vol. CE (cm^3^)	Tumor vol. FLAIR (cm^3^)	No. of distinct lesions	Surgical procedure	Histology (grade)	p53	ATRX	GFAP	KI-67	MGMT methylation	IDH-1 status	OS (wks)
1	71	F	23.4	67.8	1	Resection	GBL (WHO IV)	20%	+	+	10%	+	–	40
2	58	M	4.4	10.2	1	Biopsy	GBL (WHO IV)	15%	+	+	20%	N/A	+	76
3	56	M	25.0	111.6	1	Resection	GBL (WHO IV)	5%	+	+	25%	+	–	214
4	61	M	1.1	10.9	1	Resection	AA (WHO III)	5%	–	+	30%	+	–	71
5	73	F	61.4	130.2	1	Biopsy	GBL (WHO IV)	5–10%	+	+	7%	N/A	–	21
6	77	M	56.1	184.0	1	Resection	GBL (WHO IV)	0	+	+	15%	+	–	16
7	79	M	79.1	185.4	1	Resection	GBL (WHO IV)	10%	–	+	20%	+	–	26
8	69	F	1.6	50.1	5	Biopsy	AA (WHO III)	5%	+	+	3%	N/A	–	13
9	64	M	2.6	91.1	3	Biopsy	GBL (WHO IV)	10%	+	+	35%	N/A	–	21
10	81	M	34.4	98.1	4	Biopsy	AO (WHO III)	0	+	++	15%	N/A	+	10
11	65	M	30.3	136.5	1	Resection	GBL (WHO IV)	0	+	+	15%	+	–	44
12	77	M	5.4	23.4	1	Biopsy	GBL (WHO IV)	N/A	+	N/A	5%	N/A	–	13
13	41	M	0.0	146.5	1	Biopsy	AA (WHO III)	3%	+	+	10%	+	+	10
14	64	M	53.0	70.8	1	Biopsy	AA (WHO III)	<1%	+	–	20%	–	–	8
15	61	M	26.8	102.9	1	Resection	GBL (WHO IV)	40%	+	+	40%	N/A	–	88
16	57	F	8.0	18.8	1	Biopsy	GBL (WHO IV)	<5%	+	+	5%	N/A	–	222
17	44	F	16.8	162.3	1	Biopsy	GBL (WHO IV)	70%	–	+	25%	N/A	–	9

*Immunohistochemical analysis displayed was performed on a single sample considered as the most representative by the referring neuropathologist.*

A single contrast-enhancing (CE) lesion was detected in 13/17 patients; 3/17 patients harbored multiple CE lesions, while in one patient no definite CE tumor core was confidently identifiable. The average cumulative CE-tumor volume (per patient) was 25.3 ± 24.4 cm^3^ (range 0–79.1 cm^3^). Mean cumulative FLAIR-tumor volume (per patient) was 94.2 ± 58.7 cm^3^ (range 10.2–185.4 cm^3^). The mean rate between CE-tumor and FLAIR-tumor volume was 0.30 ± 0.20 (range 0–0.7).

Maximum safe resection was performed in 7/17, while a stereotactic biopsy was obtained in 10/17 patients. Four out of individuals harbored an anaplastic astrocytoma (WHO grade III), one had an anaplastic oligodendroglioma (1p/19q co-deleted, WHO grade III) and 12/17 patients had grade IV glioblastoma, according to WHO 2016 classification (Louis et al., 2016). Three tumors retained an IDH-1 mutation. Histopathological and molecular data are summarized in Table 1.

Mean overall survival (Table 1) was 53.1 ± 67 weeks (median 21 weeks, range 8–222 weeks). Two patients were still alive at the last follow-up assessment (49 and 51 months, respectively). The remaining 15 patients died during follow-up due to causes directly (e.g., tumor progression increasing mass effect on cerebral structures) or indirectly (e.g., aspiration pneumonia) associated with the disease.

### Map Selection

The ideal number of maps to be included simultaneously in the algorithm was thoroughly evaluated. The possible complementary information gathered by adding a higher number of maps had to be balanced with the exponential increase in the number of significantly smaller and fragmented clusters, resulting in an unreliable surgical targeting and histological correlation. For the aforementioned reasons, after examining the possible complementarity of the maps, “8” (from the combination of three different parametric maps) was identified as the ideal number of clusters for the purpose of the project. Among the available parametric perfusion maps, the intra-vascular plasma volume derived from DCE-MRI, that quantifies tumor neoangiogenesis similarly to rCBV (Alcaide-Leon et al., 2015), was selected as the most appropriate for the purpose of the study due to the lower sensitivity to susceptibility artifacts and to the presence of intralesional hemorrhagic foci, as well as for the better capability to discriminate surrounding macro-vessel from true intralesional neoangiogenic hotspots on Vp parametric maps, especially in cortical tumors growing in proximity to large vessels (Santarosa et al., 2016). Furthermore, DCE-derived Vp maps have higher spatial resolution compared to DSC-derived rCBV maps and yield an absolute quantification of perfusion parameters (Anzalone et al., 2018). Vp also allows a more informative representation of the FLAIR-tumor volume outside of the CE region compared to Ktrans (Nguyen et al., 2016; Zhang et al., 2017; Anzalone et al., 2018).

Positron emission tomography (PET) with the ^18^F-flouroazomycin arabinoside (^18^F-FAZA) radiotracer has been previously used for the non-invasive assessment of tumor hypoxia (Mapelli et al., 2021), and was chosen due to its high perfusion, high biological half-life time and fast clearance from blood, with subsequent improved tumor-to-background ratio (Mapelli and Picchio, 2020).

Among the DTI-derived maps, MD was selected as one of the simplest and most validated parameters in radiomics to distinguish tumor grade and cellular proliferation in brain gliomas (Miloushev et al., 2015). MD quantifies water molecular mobility, averaged over all the obtained directions, within tissues impeded by cell membranes and tortuosity of the extracellular space (Maier et al., 2010). This parameter is equivalent to the apparent diffusion coefficient (ADC) generated from DWI acquisitions and shows a negative correlation with tissue cellularity, as previously reported in literature (Ellingson et al., 2010; Maier et al., 2010; Chen et al., 2013; Sanvito et al., 2021).

### Habitats’ Analysis

Eight habitats were generated from cluster intersection by combining all possible HIGH (H) and LOW (L) intensity regions of each map (Vp + ^18^F-FAZA + MD) and the final tumor habitat map was composed (Figure 1).

**FIGURE 2:**
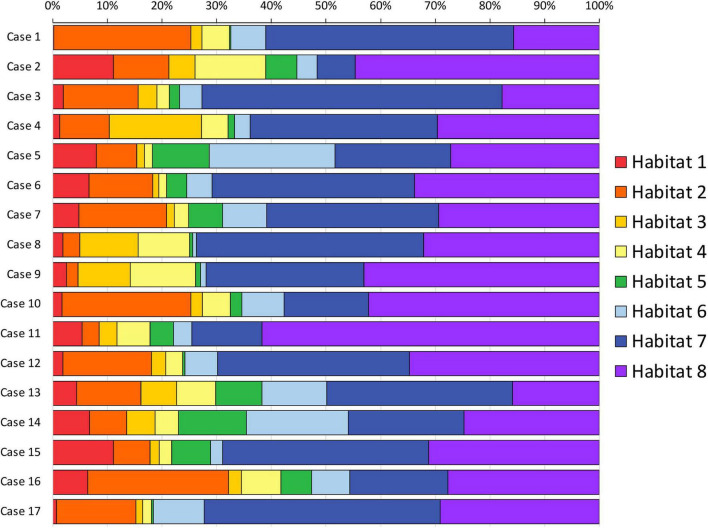
Habitats volumetric representation. The relative volumetric representation of each habitat (expressed as a percentage of the whole FLAIR-tumor volume) is reported for each case included in the series.

**FIGURE 3:**
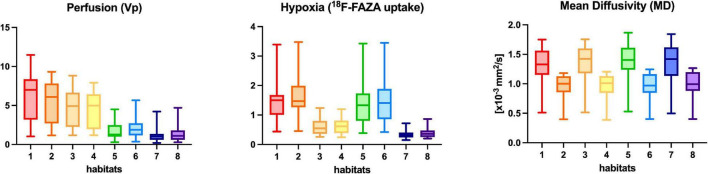
Perfusion (Vp), ^18^F-FAZA, and Diffusivity (MD) quantitative values within each habitat for the whole group of patients. Boxplots shows the distribution of mean values of Vp, ^18^F-FAZA, and MD parametric maps in each environment.

In the preliminary analysis of our experiment, we investigated the application of the same clustering algorithm to either the CE- or FLAIR-tumor, since both of them have been considered for habitat imaging studies previously published, and we assessed the obtained habitats. Clusters obtained from CE-tumor volume alone were considered too small and fragmented in order to be reliably correlated to histology, and therefore they were not deemed suitable for the purpose of the present study (Figure 1H). On the contrary, habitats obtained from FLAIR-tumor volume (which included the CE region along with the surrounding FLAIR hyperintensity) were able to divide the whole tumor in a more reproducible way, with a larger cluster amenable to a reliable histological correlation with the bioptic samples obtained (Figure 1I). For these reasons, we selected FLAIR-tumor as the final tumor mask to be investigated and all the results presented in our paper are based on its analysis. The radiological characteristics of the 8 clusters, and their volumetric representation, are summarized in Table 2 and depicted in Figure 2. Median voxel values among different maps (Vp, ^18^F-FAZA, and MD) and Habitats (1–8) are reported in Table 3. Boxplots of the quantitative distribution of mean values of Vp, ^18^F-FAZA and MD parametric maps in each environment are shown in Figure 3.

**TABLE 2:** Habitats’ characteristics and volumetric representation.

Habitats’ characteristics				Habitats representation among cases (% of total tumor volume)																	
*n*	Perfusion (Vp)	Hypoxia (FAZA)	Diffusivity (MD)	Patient number																	Row Aver.
				1	2	3	4	5	6	7	8	9	10	11	12	13	14	15	16	17	
**1**	**High**	**High**	**High**	0.2	11.1	2.0	1.3	8.0	6.7	4.8	1.9	2.6	1.7	5.4	1.9	4.4	6.7	11.1	6.4	0.7	4.5
**2**	**High**	**High**	*Low*	25.1	10.2	13.6	9.1	7.4	11.6	16.0	3.1	2.1	23.6	3.1	16.2	11.7	6.8	6.7	25.8	14.5	12.1
**3**	**High**	*Low*	**High**	2.0	4.8	3.5	16.8	1.4	1.1	1.4	10.6	9.5	2.1	3.3	2.6	6.6	5.2	1.7	2.4	1.3	4.5
**4**	**High**	*Low*	*Low*	5.0	12.9	2.2	4.9	1.5	1.4	2.6	9.4	12.0	5.1	6.0	3.1	7.1	4.3	2.3	7.2	1.6	5.2
**5**	*Low*	**High**	**High**	0.3	5.7	1.9	1.2	10.4	3.7	6.2	0.6	0.9	2.1	4.3	0.4	8.5	12.4	7.1	5.6	0.3	4.2
**6**	*Low*	**High**	*Low*	6.3	3.7	4.1	2.8	23.0	4.6	8.1	0.7	1.0	7.7	3.4	5.9	11.8	18.6	2.2	7.0	9.3	7.1
**7**	*Low*	*Low*	**High**	45.3	7.0	54.8	34.3	21.2	37.0	31.4	41.5	28.9	15.4	12.8	35.1	33.9	21.1	37.7	18.0	43.2	30.5
**8**	*Low*	*Low*	*Low*	15.7	44.7	17.8	29.6	27.2	33.8	29.3	32.1	43.1	42.2	61.7	34.7	15.9	24.7	31.2	27.7	29.1	31.8

*Aver, average row value; Diffus, diffusivity; FAZA, ^18^F-FAZA; Perfus, perfusion.*

**TABLE 3:** Plasma volume (Vp), ^18^F-labeled fluoroazomycinarabinoside (^18^F-FAZA), and mean diffusivity (MD) voxel median values among different habitats.

Map	Habitat*	Case 1	Case 2	Case 3	Case 4	Case 5	Case 6	Case 7	Case 8	Case 9	Case 10	Case 11	Case 12	Case 13	Case 14	Case 15	Case 16	Case 17	Row average

**Vp**	1-**H**HH	5.90	8.80	3.72	0.98	7.67	7.04	5.85	2.86	6.42	2.32	4.90	2.00	1.28	9.25	7.51	6.60	5.95	5.24
	2-**H**HL	5.39	8.43	3.40	1.16	5.14	5.15	6.55	2.63	5.36	2.27	4.47	1.91	1.36	7.48	8.45	6.71	6.67	4.85
	3-**H**LH	4.79	8.16	2.66	1.22	5.20	4.37	5.45	1.72	3.14	2.07	3.52	1.75	1.08	7.80	5.95	5.91	4.77	4.09
	4-**H**LL	4.74	7.46	2.84	1.10	4.82	4.41	5.25	1.69	2.97	1.91	3.25	1.55	1.10	7.04	6.18	6.12	4.59	3.94
	5-**L**HH	2.65	4.90	1.28	0.68	2.18	0.92	2.48	1.13	1.21	0.97	1.22	0.65	0.24	2.40	2.69	3.06	2.36	1.82
	6-**L**HL	2.79	5.76	1.45	0.71	2.71	2.08	2.96	1.20	1.16	1.28	1.26	0.71	0.34	2.47	2.96	3.71	2.86	2.14
	7-**L**LH	0.86	4.44	0.64	0.51	1.26	0.01	1.17	1.03	1.03	0.65	0.94	0.11	0.18	1.77	0.47	1.30	1.16	1.03
	8-**L**LL	1.66	4.80	0.66	0.47	1.75	0.15	1.46	0.99	1.02	0.91	1.07	0.21	0.33	1.92	0.84	2.15	1.72	1.30
	**Whole tumor mask**	1.94	5.89	0.83	0.64	2.32	0.25	1.88	1.14	1.40	1.14	1.23	0.31	0.43	2.81	1.15	3.84	1.67	1.70
**^18^F-FAZA**	1-H**H**H	0.77	1.55	1.26	0.73	1.97	1.73	2.08	1.38	1.06	1.52	0.84	1.38	0.41	3.45	1.48	1.43	1.05	1.42
	2-H**H**L	1.00	1.26	1.86	1.13	1.95	1.87	2.48	1.14	1.90	1.76	0.83	2.00	0.44	3.45	1.38	1.48	1.38	1.61
	3-H**L**H	0.30	0.70	0.69	0.33	1.03	0.81	1.04	0.42	0.37	0.61	0.31	0.46	0.26	1.28	0.54	0.84	0.47	0.62
	4-H**L**L	0.45	0.70	0.65	0.34	1.12	0.88	0.99	0.39	0.37	0.65	0.30	0.78	0.26	1.21	0.57	0.83	0.66	0.66
	5-L**H**H	0.73	1.69	1.64	0.66	1.81	1.66	1.92	0.74	0.69	1.76	0.64	1.22	0.37	3.46	1.33	1.27	0.87	1.32
	6-L**H**L	0.84	1.29	1.97	0.77	1.75	2.21	2.09	0.73	0.88	1.68	0.65	1.52	0.40	3.44	1.15	1.38	0.97	1.40
	7-L**L**H	0.13	0.43	0.12	0.27	0.56	0.16	0.23	0.37	0.33	0.31	0.25	0.22	0.25	0.44	0.15	0.40	0.30	0.29
	8-L**L**L	0.22	0.46	0.16	0.26	0.88	0.21	0.28	0.36	0.32	0.33	0.20	0.29	0.26	0.56	0.19	0.56	0.45	0.35
	**Whole tumor mask**	0.24	0.63	0.18	0.31	1.31	0.25	0.47	0.38	0.34	0.45	0.24	0.35	0.30	1.79	0.26	0.92	0.42	0.52
**MD^§^**	1-HH**H**	1.60	1.05	1.54	1.22	0.97	1.49	1.64	0.49	1.26	1.24	1.45	1.28	1.48	1.46	1.70	1.24	0.95	1.30
	2-HH**L**	0.99	0.85	1.13	0.86	0.81	1.13	1.15	0.40	1.03	0.90	1.08	0.95	1.19	1.12	1.20	0.88	0.76	0.97
	3-HL**H**	1.68	1.05	1.65	1.33	1.04	1.45	1.65	0.50	1.20	1.30	1.40	1.39	1.51	1.46	1.64	1.27	0.99	1.32
	4-HL**L**	1.10	0.79	1.15	1.02	0.73	1.14	1.23	0.40	0.98	0.93	1.01	1.03	1.21	1.15	1.20	0.89	0.81	0.99
	5-LH**H**	1.51	1.28	1.61	1.20	0.95	1.57	1.70	0.51	1.23	1.29	1.71	1.27	1.50	1.42	1.84	1.31	0.95	1.34
	6-LH**L**	0.98	0.83	1.25	0.89	0.78	1.09	1.21	0.41	0.98	0.92	1.04	0.92	1.22	1.15	1.30	0.97	0.71	0.98
	7-LL**H**	1.85	1.04	1.72	1.26	1.01	1.53	1.63	0.49	1.17	1.36	1.40	1.37	1.56	1.46	1.61	1.31	0.99	1.34
	8-LL**L**	1.22	0.78	1.30	1.00	0.72	1.15	1.27	0.41	1.00	0.95	0.96	1.06	1.26	1.13	1.23	0.97	0.83	1.01
	**Whole tumor mask**	1.41	0.84	1.58	1.14	0.84	1.33	1.39	0.46	1.07	0.97	1.02	1.13	1.41	1.27	1.50	1.01	0.88	1.13

*“H” and “L” correspond, respectively, to the High and Low-intensity cluster of the considered parametric maps.

*^§^Median values are here reported with a multiplication factor of 10^3^.*

**FIGURE 4:**
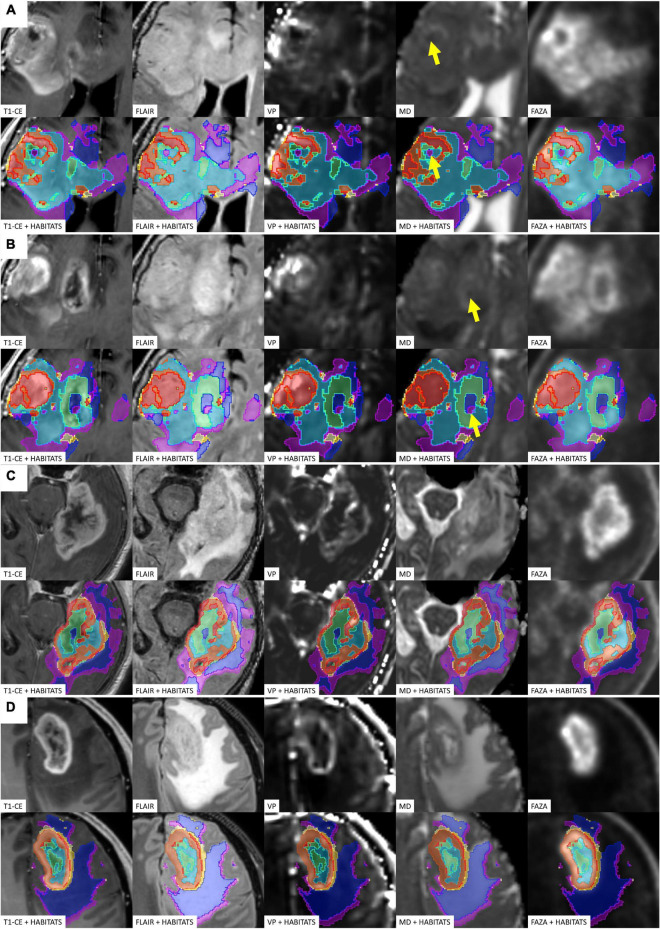
Four different illustrative cases **(A–D)** showing habitats distribution and reproducibility of spatial habitat imaging maps. A table listing the key colors (1–red, 2–orange, 3–gold, 4–yellow, 5–aquamarine, 6–light blue, 7–dark blue, and 8–violet) assigned to each habitat is reported in Figure 5. The yellow arrows shows point areas of microhemorragic foci resulting in a MD signal restriction.

**FIGURE 5:**
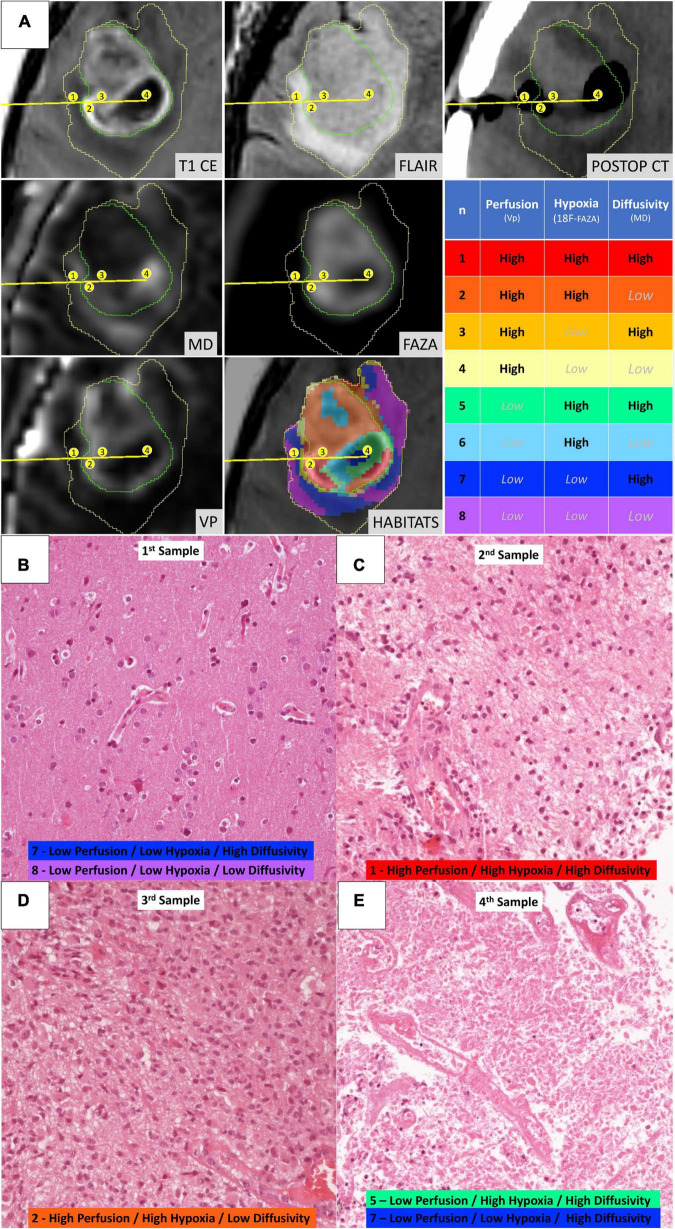
Illustrative case of a 57-year-old female patient (case 16 in Table 1) who underwent stereotactic biopsy. **(A)** The trajectory path (yellow line) and the four consecutive sampling sites (enumerated in the yellow circles) are displayed in axial formatted pre-operative T1-CE, FLAIR, MD, ^18^F-FAZA, Vp, and post-operative CT images; the spatial distribution of the identified habitats and the assigned key colors are also displayed (1–red, 2–orange, 3–gold, 4–yellow, 5–aquamarine, 6–light blue, 7–dark blue, and 8–violet). **(B)** Microscopic slide [20× magnification, Hematoxylin and Eosin (H&E)] from the first sampling (corresponding to Habitat 7–Low Perfusion/Low Hypoxia/High Diffusivity and Habitat 8–Low Perfusion/Low Hypoxia/Low Diffusivity) showing a cortical/subcortical area with normal tissue architecture, presence of normal vessel and no significant increase in cellularity, but rather mild reactive gliosis. **(C)** Microscopic slide (20× magnification, H&E) from the second sampling (Habitat 1–High Perfusion/High Hypoxia/High Diffusivity) showing a clearly pathological area of white matter with increased cellularity, disruption of the physiological tissue architecture, and abnormal vessels. **(D)** Microscopic slide (20× magnification, H&E) from the third sampling (Habitat 2–High Perfusion/High Hypoxia/Low Diffusivity) showing neoplastic tissue with cytoarchitecture disruption, elevated cellularity with the presence of gemistocytic astrocytes, nuclear atypia, abundant eosinophilic cytoplasm, abnormal vascularization. **(E)** Microscopic slide (20× magnification, H&E) from the fourth sampling (Habitat 5–Low Perfusion/High Hypoxia/High Diffusivity and Habitat 7–Low Perfusion/Low Hypoxia/High Diffusivity) showing tissue with extensive necrosis.

**FIGURE 6:**
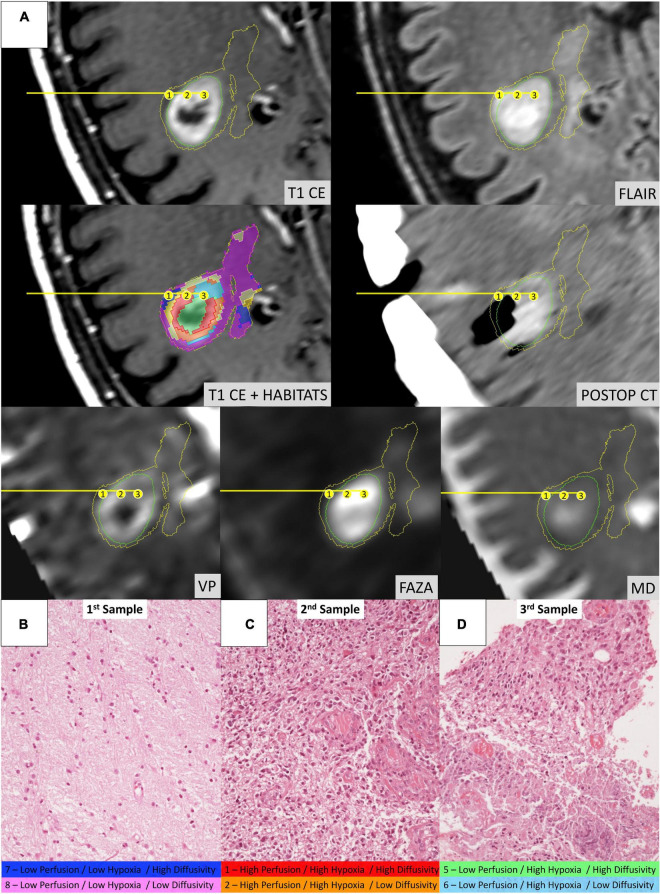
Illustrative case of a 58-year-old male patient (Case 2 in Table 1) who underwent stereotactic biopsy. **(A)** The trajectory path (yellow line) and the three consecutive sampling sites (enumerated in the yellow circles) are displayed in axial formatted pre-operative T1-CE, FLAIR, MD, ^18^F-FAZA, Vp, and post-operative CT images; habitats were identified using the same key-color described in Figure 5. **(B)** Microscopic slide [20× magnification, Hematoxylin and Eosin (H&E)] from the first sampling (Habitat 7–Low Perfusion/Low Hypoxia/High Diffusivity and Habitat 8–Low Perfusion/Low Hypoxia/Low Diffusivity) showing a seemingly normal white matter architecture. **(C)** Microscopic slide (20× magnification, H&E) from the second sampling (Habitat 1–High Perfusion/High Hypoxia/High Diffusivity and Habitat 2–High Perfusion/High Hypoxia/Low Diffusivity) showing evident hypercellularity with nuclear atypias and marked vascular proliferation. **(D)** Microscopic slide (20× magnification, H&E) from the third sampling (Habitat 5–Low Perfusion/High Hypoxia/High Diffusivity and Habitat 6–Low Perfusion/High Hypoxia/Low Diffusivity) areas of vital tumoral tissue (on the upper part) mixed with areas of necrosis (on the lower part).

**FIGURE 7:**
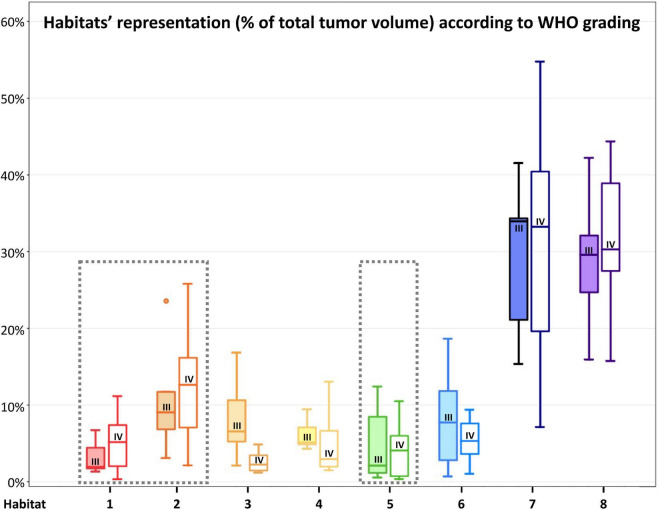
Habitats’ representation (% of total tumor volume) according to WHO 2016 grade. For each habitat, the boxplot on the left (with filled color) represents WHO grade III tumors, while the boxplot on the right (not filled with color) represents WHO grade IV tumors. WHO grade IV gliomas had a higher (although not statistically significant) representation of Habitat 1 and 2 (considered the most aggressive clusters from a metabolic point of view) and Habitat 5 (mostly associated with necrotic areas). The most aggressive clusters are surrounded by a gray box with a dashed line.

**FIGURE 8:**
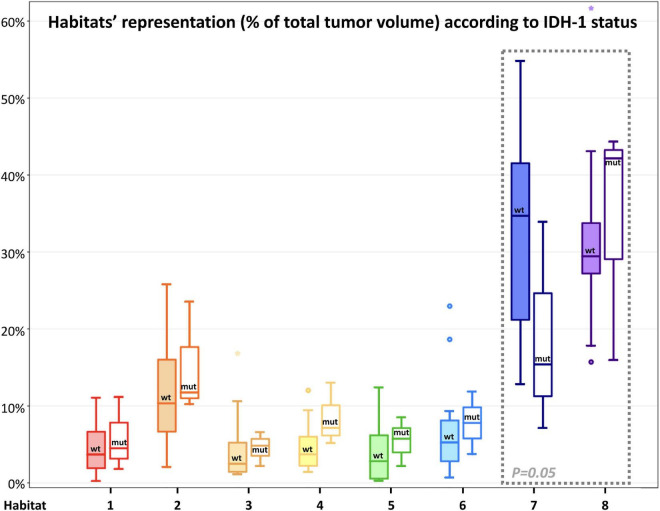
Habitats’ representation (% of total tumor volume) according to IDH-1 status. For each habitat, the boxplot on the left (with filled color) represents IDH-1 Wild-type tumors, while the boxplot on the right (not filled with color) represents IDH-1 mutated tumors. Main differences were found in the representation of Habitat 7 (possibly embodying pure vasogenic edema) and 8 (possibly representing infiltrative edema). The former was significantly (*P* = 0.05) higher in IDH wild-type tumors, while the latter was higher (not significantly) in IDH-mutated lesions; these latter two habitats were surrounded by a gray box with a dashed line to highlight the differences.

Figure 4 depicts habitats distribution in four different cases of the present series. Figures 5, 6 present two illustrative cases showing the consistency between the functional spatial tumor habitats and the microscopic morphology of the corresponding tissue. The microscopic semi-quantitative analysis (cellularity, extent of necrosis, number of hyperplastic vessels) of each sample and the corresponding Habitats on imaging are reported in Table 4. Table 5 and Figures 7, 8 report habitats’ prevalence according to grade and molecular features. Habitats’ descriptions and characteristics are analyzed below.

**TABLE 4:** Semi-quantitative histopathologic analysis for each bioptic sample obtained.

Case number	Sample	Cellularity*^a^* (1–5)	Class of necrosis*^b^* (0–5)	Number of hyperplastic vessels counted (*n*)	Habitats involved (ordered by prevalence inside VOI)*^c^*
2	A	1	0	0	7, 8
	B	4	0	43	2
	C	4	1	21	5
5	A	3	2	1	6
	B	4	4	1	2, 5
	C	3	5	3	5, 7, 8
	D§	3	3	6	5, 6
8	A	2	0	0	1
	B	2	0	0	1
	C	2	0	1	1
	D	2	0	0	1
9	A	5	2	20	1
	B	5	2	19	1
10	A	1	0	0	7, 8
	B	3	1	8	2
12	A	1	0	4	8, 2, 6
	B	3	2	4	2, 6
	C	4	1	8	2
13	A	1	0	0	7
	B	1	0	0	5
	C	3	0	0	3
	D	3	0	2	1,3
14	A	3	0	5	7,8,6
	B	4	0	11	6
16	A	1	0	0	7, 8
	B	3	0	28	1
	C	5	1	11	2
	D	N/A*	5	N/A*	5, 7
17	A	4	0	6	2
	B	4	1	7	2
	C	4	0	4	6, 2

*Features assessed in the present analysis were: cellularity, extent of necrosis, and number of hyperplastic vessels counted.*

*To address the issue of length variability among different bioptic specimens and to maximize the correspondence with the target point set on the stereotactic planning system, only the central portion (extending 5 mm along the greatest axis of the specimen in its largest section available) was analyzed in each sample.*

*The corresponding spatial tumor imaging habitats involved are reported in the last column, ordered accordingly to their relative prevalence inside of the 5 mm diameter volume of interest.*

*N/A, not applicable.*

*^a^Cellularity was graded with a score from 1 (seemingly normal representation) to 5 (extremely high cellular density).*

*^b^Classes considered for necrosis (expressed as a rate over the whole area considered for each specimen) were the following: 0 (0%), 1 (≤10%), 2 (11–25%), 3 (26–50%), 4 (51–75%), 5 (76–100%).*

*^c^A 5 mm diameter volume of interest (VOI) was drawn according to the exact stereotactic coordinates of each target and needle window orientation, then carefully verified also in the coregistered in the early post-operative CT scan. In this column, the included habitats are reported. Since the spatial habitat maps were not available at the time of planning, final sampling VOI ended at the intersection of multiple clusters in many cases; in the latter case, clusters are listed in order of maximum volumetric representation inside of the VOI.*

*§This sample was only 4 mm long.*

*It was not possible to calculate these features since the sample was completely represented by necrosis.

**TABLE 5:** Mean and median representation of the eight different habitats according to IDH-1 status and WHO 2016 grade.

Habitats characteristics				Habitats’ representation (% of total tumor volume) according to							
				IDH-1 status				WHO 2016 grading			
***n***	**Perfusion** **(Vp)**	**Hypoxia (^18^F-FAZA)**	**Diffusivity (MD)**	**Wild-type**		**Mutated**		**III**		**IV**	
				**Mean (%)**	**Median (%)**	**Mean (%)**	**Median (%)**	**Mean (%)**	**Median (%)**	**Mean (%)**	**Median (%)**

**1**	**High**	**High**	**High**	4.3	3.7	5.8	4.4	3.2	1.9	5.1	5.1
**2**	**High**	**High**	*Low*	11.5	10.3	15.2	11.7	10.9	9.1	12.7	12.6
**3**	**High**	*Low*	**High**	4.5	2.5	4.5	4.8	8.3	6.6	2.9	2.2
**4**	**High**	*Low*	*Low*	4.6	3.7	8.4	7.1	6.2	5.1	4.8	2.9
**5**	*Low*	**High**	**High**	4.0	2.8	5.4	5.7	4.9	2.1	3.9	4.0
**6**	*Low*	**High**	*Low*	6.9	5.3	7.8	7.7	8.3	7.7	6.6	5.3
**7**	*Low*	*Low*	**High**	33.0	34.7	18.8	15.4	29.3	33.9	31.0	33.2
**8**	*Low*	*Low*	*Low*	31.3	29.5	34.2	42.2	28.9	29.6	33.0	30.3

*Data are expressed as a percentage of the cumulative tumor volume of the whole series.*

**Habitat 1**, characterized by high perfusion (the highest Vp values among all clusters, Figure 3), high hypoxia, and high diffusivity, corresponded to areas of mild hyperintensity on T2/FLAIR sequences and intense enhancement on T1–CE sequences; Habitat 1 voxels were localized inside of CE-tumor segmentation in 92% of the cases overall. No areas of necrosis were identifiable on cMRI sequences inside this cluster. This cluster was often located at the periphery of the CE-tumor, leaving other aggressive clusters more internally in the tumor core. **Habitat 2** was denoted by elevated perfusion, hypoxia, and restricted diffusion, representing, alone, about half of CE-tumor volume overall. On cMRI it was denoted by a mild hyperintensity on T2/FLAIR sequences and no visible necrosis. Similarly to Habitat 1, this cluster was localized inside of CE-tumor segmentation in 89% of the cases overall, usually in close proximity to the previous one; however, when compared to the first cluster, Habitat 2 was much more represented (12.1% vs. 4.5%), harboring a less intense contrast enhancement on T1-CE sequences, lower perfusion values, more reduced diffusivity (possibly reflecting higher cellularity) and, generally (in 12/17 patients), higher hypoxia (^18^F-FAZA-uptake). At histopathology these first two habitats were largely associated with a higher number of hyperplastic vessels and cellularity, whereas a very low rate of necrosis. Moreover, their relative prevalence was higher among WHO grade IV gliomas.

**Habitats 3** and **4**, both sharing low hypoxia and high perfusion, while a different degree of diffusivity, were extremely fragmented and poorly represented among all cases (4.5 and 5.2% of the tumor volume, respectively). They were mostly grouped together, often located either at the interface between tumor CE core and the surrounding area of FLAIR-hyperintensity with no CE, adjacent to large vessels or close to the cerebral cortex. Only about 25% of the voxels of these clusters were located inside of CE-tumor segmentation.

**Habitat 5** was characterized by high hypoxia, low perfusion, and increased diffusivity (possibly denoting low cellularity). It was located inside of CE-tumor segmentation in 93% of cases, characterized by necrotic areas at both cMRI and histopathology. This was the least prevalent habitat, with an average representation of 4.2% (range 0.3–12.4%) among the considered tumors; however, it appeared more represented in WHO grade IV (4%) compared to WHO grade III tumors (2.1%).

**Habitat 6** (low perfusion, reduced diffusivity, and high hypoxia) had a relatively high representation compared to the aforementioned clusters (average 7.1%, range 0.7–23%), being mostly localized inside of CE-tumor segmentation (75% of the cases). It was predominantly identified as areas of less intense CE inside the tumor core, being closely related to Habitat 5 (located more internally) and Habitat 2 (located more externally), as well perceivable in Figures 4C,D. Conversely, in different cases, this cluster was identified as areas of faint contrast-enhancement outside the main tumor cores, as one could appreciate in Figures 4A,B. At histopathology this habitat showed features of intermediate aggressiveness.

The remaining two clusters had the largest volumetric representation (each one representing over 30% of the total FLAIR-tumor volume) and were both found in areas with no CE in roughly 95% of the cases. **Habitat 7** was, possibly, the least aggressive one inside the tumor mask due to its low values in all the considered parameters; it was characterized by the highest absolute MD values among all clusters (possibly reflecting the lowest cellularity) and it was located in areas of markedly hyperintense FLAIR signal (Figures 4B,D). The distribution of Habitat 7 was significantly higher (mean 33.3% vs. 18.8%, median 34.7% vs. 15.4% *P* = 0.05, independent sampled Mann-Whitney *U* test) in IDH-1 wild-type tumor compared to IDH-1 mutated (see Table 5 and Figure 8). **Habitat 8** was characterized, on the contrary, by reduced diffusivity; moreover, perfusion values and hypoxia values tended to be higher than those of Habitat 7 (Table 3). **Habitat 8** was also identified in the setting of hemorrhagic foci or necrotic areas with micro-hemorrhagic components (as identified on susceptibility weighted imaging, SWI) or viscous mucinous components, due to low MD values resulting from extravascular blood components or liquefactive necrosis (Kang et al., 2001; Chen et al., 2018; Ono et al., 2018) (yellow arrow on Figures 4A,B). Additionally, as easily noticeable in Figures 4C,D, the localization of Habitat 8 in cortical areas distant from the tumor core is likely related to the lower MD values of the cerebral cortex, compared to underlying edematous white matter. When considering bioptic samples obtained outside the CE-tumor these last two habitats were characterized by histological features of low cellularity and no signs of necrosis or neovascularization. Conversely, they showed extensive necrosis with no viable tissue, in those cases biopsied inside the CE-tumor.

## Discussion

In this study, quantitative radiomic parameters from PET and MR images were derived for the assessment of hypoxia, perfusion, and tissue diffusion in a cohort of high-grade glioma patients, and a pipeline for a voxel-wise analysis to combine them in a number of discrete spatial tumor habitats was implemented for the clustering of highly hypoxic areas as well as areas of similar perfusion and diffusion characteristics. By this approach, eight regions were consistently highlighted within the tumor volume, reflecting the intra-tumor heterogeneity of physiological and histopathological microenvironments. Spatial tumor habitats demonstrated high reproducibility across patients and correlation with disease-specific histopathological and cMRI features. To the best of our knowledge, this represents the first study combining the aforementioned parameters to define tumor habitats.

### Combined PET and MRI Hypoxia, Perfusion, and Diffusion Spatial Habitats’ Features

Of the eight habitats identified, we might speculate that the first two could identify the “vital” core of the tumor, with the second cluster being, hypothetically, the most aggressive component. Interestingly, **Habitat 1**, characterized by the highest Vp values (Figure 3), was often located more peripherally than **Habitat 2**, which is in line with the fact that perfusion values tend to be higher at the periphery of the tumor core (Ponte et al., 2017). These two habitats also showed the highest hypoxia values, data in accordance with previously reported studies (Ponte et al., 2017). The fact that the tumor “vital core” represents, at the same time, the most perfused and most hypoxic area, although it might seem paradoxical, is likely related to the functionally and structurally abnormal vascularity that characterizes HGGs, unable to provide sufficient perfusion and oxygen supply (Ponte et al., 2017; Rosinska and Gavard, 2021). The higher hyperintensity on T1-weighted CE sequences noticed inside **Habitat 1** could be related to higher perfusion and/or vascular permeability, as confirmed by the significantly higher Vp, but also rCBV and Ktrans values noted in this cluster. The higher MD values of **Habitat 1**, when compared to **Habitat 2,** could imply lower tissue cellularity but also the presence of stronger vasogenic edema due to blood-brain barrier leakage. As somehow expected, the distribution of these habitats, together with the other most “aggressive” biologic cluster representing areas of tumor necrosis (Habitat 5), was higher in WHO grade IV tumors compared to WHO III.

The poor representation of **Habitat 3** and **4** might be related to the low prevalence of well-oxygenated areas inside the tumor core. Since a certain degree of hypoxia is required to trigger neoangiogenesis (and thus increased perfusion), it is unlikely to find highly perfused but scarcely hypoxic clusters within HGGs (Huang et al., 2016; Ponte et al., 2017).

**Habitat 5** represents necrotic areas still including some degree of vital tissue, since the ^18^F-FAZA-PET tracer needs hypoxic, yet vital, cells in order to be reduced to reactive oxygen radicals and remain locally trapped (Halmos et al., 2014; Mapelli and Picchio, 2020).

**Habitat 6**, when colocalizing with areas of faint contrast-enhancement outside the tumor “vital” and necrotic cores, was interpreted by the authors as new tumoral seeds progressing toward higher malignancy, since it harbored reduced diffusivity and increased hypoxia, but not yet a significantly augmented vascularization. On the contrary, when **Habitat 6** was identified inside of the tumor core, it was interpreted as regions where tumor growth exceeded neovascularization and that were close to evolving into necrosis.

Finally, we suggested that **Habitat 8**, due to its reduced diffusivity, might identify areas of tumor infiltration beyond the CE vital core, while **Habitat 7** areas of vasogenic edema (with no, or less prominent, tumor infiltration). Interestingly, in our series, the relative volume of pure edema (Habitat 7) was significantly higher in IDH-1 wild-type tumors, compared to IDH-1 mutated lesions, similarly to other recent studies (Dubinski et al., 2021).

Considering the known models of tumor progression (Holash et al., 1999; Brat et al., 2002; Hardee and Zagzag, 2012; Stadlbauer et al., 2018b), we may hypothesize a possible evolution over the time of the identified PET/MRI habitats: areas of tumor infiltration in the white matter (**Habitat 8**) might eventually develop some degree of hypoxia (**Habitat 6**), which, in turn, trigger neovascularization possibly through tumor cells secretion of mediators such as HIF-1α and VEGF (Stadlbauer et al., 2018b) and increased perfusion values, becoming a tumor “vital core”: **Habitat 1** and (perhaps subsequently) **Habitat** **2**. In due course, the tumor growth in the vital core will overwhelm its abnormal vascularization, leading toward areas in a pre-necrotic state (**Habitat 6**); as the necrosis progresses, cellularity will decrease (**Habitat 5**) eventually turning into liquefactive necrosis, with no remaining vital cells and showing, consequently, a concomitant drop in hypoxia signaling and ^18^F-FAZA-tracer uptake (**Habitat 7**). The aforementioned “habitat progression” is easily perceivable in Figures 4C,D, where a somehow concentric distribution of the clusters is identifiable.

### Potential Clinical Applications

Advanced neuroimaging techniques such as PWI, dMRI, or PET with different radiotracers are usually evaluated independently in the clinics or are rather combined into an average parameter for the entire region of interest, discarding important spatial information (O’Connor et al., 2015; Napel et al., 2018). The simultaneous combination of diverse imaging modalities into a single habitat map leverages the information of the individual imaging datasets, possibly enhancing the ability to capture subtle physiological differences within tumor tissue and better revealing the complexity of spatial and temporal heterogeneity, both at initial diagnosis and during follow-up (Yip et al., 2017; Napel et al., 2018).

Furthermore, the heterogeneous biology of HGGs challenges the reliability of limited needle biopsy sampling for making disease-wide characterization. Nevertheless, needle biopsies are utilized, to date, in about a third of cases to define HGG diagnosis. The rate of non-diagnostic (to be repeated) stereotactic biopsies is 7–10% (Lu et al., 2015; Sciortino et al., 2019), and complete diagnostic accuracy has been reported as low as 33–64% of cases (compared to pathologic analysis obtained with the subsequent gross-total resection of the same lesion) (Jackson et al., 2001; Muragaki et al., 2008; Reithmeier et al., 2013). Integrating spatial tumor habitats into a bioptic planning system might better guide the neurosurgeon in sampling the most relevant functional regions of the tumor, especially by virtue of the much easier perception/understanding of the colored maps of a limited number of habitats, compared to the thorough expertise in advanced neuroimaging needed to interpret multiple coregistered parametric maps elaborated from PWI, dMRI, or PET radiotracers (as depicted in the illustrative cases of Figures 5, 6). This might be particularly valuable in those gliomas showing poor or no enhancement on T1-CE sequences, since about one-third of non-enhancing gliomas contain aggressive areas that, if correctly included in the pathologic analysis, may lead to classify the lesion into a higher-grade tumor, with consequently remarkable differences on treatment management and prognosis. In these cases, the few samples achievable *via* needle biopsy might easily underestimate the actual tumor grade if not adequately guided to the most aggressive areas.

We believe that further exploration of hypoxia-, perfusion-, and diffusion-derived habitats might provide precious, holistic information about intra-tumor heterogeneity, unveiling regional diversity both at the level of DNA mutation and gene expression profiles, helping in prognosis stratification and, perhaps, in future, treatment selection. The precise identification of the areas with the highest perfusion, microstructural alteration and cellularity, and hypoxia inside and outside the tumor contrast enhancement component could represent an enormous aid in surgical and radiotherapy planning. In the former case, the identification of an aggressive habitat might induce the surgeon to seek its maximum safe resection rate even if not showing evident contrast enhancement. In the latter case, radiation dose painting with boosts on the most aggressive and resistant clusters represents one of the most appealing perspectives for the next future (Castellano et al., 2021). Finally, the identified habitats could represent a precious tool in monitoring the dynamical spatio-temporal modification in glioma heterogeneity during therapies and follow-up and could identify more clearly areas of tumor response or progression.

### Limitations and Future Work

One main drawback of the present work is the limited number of cases analyzed and their heterogeneity (e.g., tumor histology, grading, molecular characteristics, volume, location, patient’s age, comorbidities, type of surgery) which have likely hampered the achievement of conclusive associations between the variables involved (parametric map values, habitats, histopathological data, and patient outcome/survival).

In the subgroup of 10 patients undergoing a stereotactic biopsy, we were able to identify a qualitative correlation between the expected microenvironment of the different habitats and the actual histopathologic morphology, although only a preliminary analysis was possible in this small and heterogenous series and no definitive, statistically significant, conclusion could be drawn. The designation of biopsy target spots and path was planned in order to maximize patient safety while ensuring final diagnosis, according to the best clinical practice, by means of the minimum amount of sampling deemed necessary, due to ethical concerns. This, along with the fact that the spatial tumor habitats were not available to the neurosurgeon pre-operatively, prevented the acquisition of a sufficient variety of sampled habitats in the same lesion and frequently ended in sampling over multiple clusters at once, therefore limiting the possibility of direct associations. A thorough evaluation of prospectively and systematically collected data is clearly necessary to correlate the identified clusters with histopathological, immunohistochemical, and molecular biomarkers.

Multiparametric analysis on a per-voxel basis to generate habitats maps remains challenging, because this requires spatial registration of all imaging sequences, which can be hampered by the resolution differences and deformations that can occur between different acquisition modalities (Winfield et al., 2016; Napel et al., 2018). In the future, the use of a fully hybrid PET/MR scanner could further enhance the quality and spatial discrimination of the data obtained from dMRI and PWI while optimizing scanning time and image coregistration. Moreover, considerable processing and analyses were required initially for the generation of the final spatial tumor habitats, which demanded intensive labor and both clinical imaging scientist and informatics/engineering expertise. This requirement could be particularly critical in clinical practice when a short time in-between imaging and surgery is available. However, we believe that this could be overcome in the future *via* dedicated flow-charts with automated algorithms, providing the benefit of a much easier data interpretation for the neurosurgeon in charge of planning the procedure.

A limitation of the applied algorithm was the possible colocalization of the same radiological habitat in different tumor microenvironments. This mainly derived from the fact that MD, despite it has been proved to be inversely correlated to tissue cellularity, also depends on other parameters such as free water, perfusion/ischemia, temperature, and the presence of blood or mucinous components (Maier et al., 2010; Chen et al., 2018; Ono et al., 2018). For instance, Habitat 8 (which was thought to mainly represent areas of increased cellularity due to tumor infiltration of the surrounding white matter) was also found in proximity to microhemorrhagic foci, liquefactive necrosis including viscous mucinous components or cerebral vessels and cortex. On the other hand, perfusion values may be partly influenced by the presence of macrovessels passing through the analyzed tissue. A possible solution to the issues above could be leaving a larger margin from the aforementioned regions while segmenting tumor mask, adding/modifying the parametric maps utilized in the procedure, or identifying different cut-offs or algorithms for the clustering analysis.

The adopted Otsu algorithm is an easy-to-apply method able to find the optimal binary threshold for a single parametric map and outputting two disjoint regions, with high and low-intensity voxels. This allows combining different map subregions to obtain clusters of possible clinical significance. However, this method is limited in capturing all of the variety of tissues present in the tumoral ROI due to the net cut-off of the binary threshold, and it inherently assumes that all maps contain only two tissue classes, neglecting the fact that some maps could be sensitive to multiple tissue microenvironments. Moreover, this technique quickly leads to challenges when trying to combine multiple maps: the addition of more than 3 maps to the computation was found inadequate of the purpose of the study, due to the exponential increase of clusters number, making them excessively small and fragmented within the tumor ROI to offer a reliable correlation with bioptic specimens. The adoption of more refined data-driven clustering methods such as k-means, which allow to consider multiple parametric maps at the same time while preventing over-segmentation would have, perhaps, better addressed this particular issue. However, for the purposes of the present work, the generated clusters needed to be consistent in number and quantitative features among the different cases, in order to allow a possible biological interpretation of the individual habitats.

The alternative adoption of novel advanced MR techniques capable of assessing tissue hypoxia could represent another extremely appealing eventuality, since it would avoid the need for PET hypoxia radiotracers (such as ^18^F-FMISO or ^18^F-FAZA) that can be difficult to be gathered from specialized manufacturers or produced locally, expensive, time-consuming, and still problematic to be implemented in common practice due to the lack of a thorough clinical validation and institutional authorization. Indeed, in more recent years, resting-state blood oxygen level-dependent (BOLD) acquisitions have been successfully employed in the detection of areas of neurovascular uncoupling and hypoxia inside of tumoral tissue (Stadlbauer et al., 2011, 2018a,b; Pillai and Zaca, 2012; Englander et al., 2018). These maps could be easily acquired in the same MRI protocol together with morphological sequences, dMRI, and PWI, and can be eventually integrated into our algorithm to generate spatial tumor imaging habitats.

This study represents a preliminary analysis, as part of a feasibility study: additional future work will involve performing a systematic correlation of spatial habitat analysis with patients’ outcome, survival and recurrence, in a larger, prospective clinical series. Moreover, it would be interesting to follow-up patients over the course of the disease with the tumor habitat maps in order to monitor spatio-temporal changes and be able to validate the speculated spatio-temporal evolution of the PET/MRI tumor habitats.

## Conclusion

This study shows the feasibility of an innovative PET and MRI approach for the assessment of hypoxia, perfusion, and diffusion in HGGs to derive combined habitats for the clustering of intra-tumor heterogeneity. The high consistence and reproducibility of the proposed spatial habitat imaging approach, as well as the correlation of the identified habitats with disease-specific histopathological features suggest that this approach can be valuable to decode metabolic, structural, and physiological patterns at same moment and non-invasively, in order to identify different areas within heterogeneous tumors. A systematic, prospective validation of this combined PET and MRI hypoxia, perfusion, and diffusion spatial tumor habitat map in a larger cohort of malignant glioma patients is warranted to define its impact on individualized therapy planning, prediction of prognosis and follow-up.

## Data Availability Statement

All data generated and analysed during the current study are available from the corresponding author on reasonable request.

## Ethics Statement

The studies involving human participants were reviewed and approved by the Institutional Ethics Committee of Ospedale San Raffaele, Milan, Italy. Patients provided their written informed consent to participate in this study and for the publication of any potentially identifiable images or data included in this article.

## Author Contributions

AC and MB: conception and design of the study, imaging dataset creation and coregistration on 3D Slicer, tumor segmentation, and habitat analysis. MB and PMa: patient enrollment and clinical data collection. PS, VB, FF, NA, and AC: imaging acquisition and ^18^F-FAZA-PET, PWI, and DTI map generation. MB, NP, and AC: data management and statistical analysis. MB: map implementation in surgical planning, correlations with surgical data, and writing first draft of the manuscript. NP and LP: creation and execution of MATLAB scripts for map binarization and habitats generation. MC and MB: histopathological analysis. AC: conceived the original idea and was in charge of the overall direction and revision of the study. NA, MP, PMo, and AF: helped to supervise the project. All authors contributed to manuscript revision, read, and approved the submitted version.

## Conflict of Interest

The authors declare that the research was conducted in the absence of any commercial or financial relationships that could be construed as a potential conflict of interest.

## Publisher’s Note

All claims expressed in this article are solely those of the authors and do not necessarily represent those of their affiliated organizations, or those of the publisher, the editors and the reviewers. Any product that may be evaluated in this article, or claim that may be made by its manufacturer, is not guaranteed or endorsed by the publisher.

## Footnotes

1
https://fsl.fmrib.ox.ac.uk/

2
http://www.slicer.org

## Abbreviations

^18^F-FAZA: ^18^F-labeled fluoroazomycinarabinoside
ADC: apparent diffusion coefficient
CE: contrast-enhanced
CT: computed tomography
DCE: dynamic contrast-enhanced
DSC: dynamic susceptibility contrast-enhanced
cMRI: conventional magnetic resonance imaging
dMRI: diffusion magnetic resonance imaging techniques
DTI: diffusion tensor imaging
DWI: diffusion-weighted imaging
FLAIR: fluid-attenuated inversion recovery
HGG: high-grade glioma
HIF-1 α: hypoxia-inducible factor 1 α
IDH: isocitrate dehydrogenase
Ktrans: volume transfer constant
MD: mean diffusivity
MGMT: O6-methylguanine-DNA methyltransferase
MRI: magnetic resonance imaging
PET: positron emission tomography
PWI: perfusion-weighted magnetic resonance imaging
rCBV: relative cerebral blood volume
VEGF: vascular-endothelial growth factor
Vp: plasma volume
WHO: World Health Organization.

## References

[B1] Abdo R. A. Lamare F. Fernandez P. Bentourkia M. (2019). Analysis of hypoxia in human glioblastoma tumors with dynamic 18F-FMISO PET imaging. *Aust. Phys. Eng. Sci. Med.* 42 981–993. 10.1007/s13246-019-00797-8 31520369

[B2] Alcaide-Leon P. Pareto D. Martinez-Saez E. Auger C. Bharatha A. Rovira A. (2015). Pixel-by-pixel comparison of volume transfer constant and estimates of cerebral blood volume from dynamic contrast-enhanced and dynamic susceptibility contrast-enhanced mr imaging in high-grade gliomas. *AJNR Am. J. Neuroradiol.* 36 871–876. 10.3174/ajnr.A4231 25634715 PMC7990585

[B3] Anzalone N. Castellano A. Cadioli M. Conte G. M. Cuccarini V. Bizzi A. (2018). Brain gliomas: multicenter standardized assessment of dynamic contrast-enhanced and dynamic susceptibility contrast MR images. *Radiology* 287 933–943. 29361245 10.1148/radiol.2017170362

[B4] Barajas R. F. Jr. Phillips J. J. Parvataneni R. Molinaro A. Essock-Burns E. Bourne G. (2012). Regional variation in histopathologic features of tumor specimens from treatment-naive glioblastoma correlates with anatomic and physiologic MR Imaging. *Neuro Oncol.* 14 942–954. 10.1093/neuonc/nos128 22711606 PMC3379808

[B5] Beig N. Bera K. Prasanna P. Antunes J. Correa R. Singh S. (2020). Radiogenomic-based survival risk stratification of tumor habitat on GD-T1W MRI is associated with biological processes in glioblastoma. *Clin. Cancer Res.* 26 1866–1876. 10.1158/1078-0432.CCR-19-2556 32079590 PMC7165059

[B6] Brat D. J. Castellano-Sanchez A. Kaur B. Van Meir E. G. (2002). Genetic and biologic progression in astrocytomas and their relation to angiogenic dysregulation. *Adv. Anat. Pathol.* 9 24–36. 10.1097/00125480-200201000-00004 11756757

[B7] Castellano A. Falini A. (2016). Progress in neuro-imaging of brain tumors. *Curr. Opin. Oncol.* 28 484–493. 10.1097/CCO.0000000000000328 27649026

[B8] Castellano A. Bailo M. Cicone F. Carideo L. Quartuccio N. Mortini P. (2021). Advanced imaging techniques for radiotherapy planning of Gliomas. *Cancers (Basel)* 13:1063. 10.3390/cancers13051063 33802292 PMC7959155

[B9] Chen L. Liu M. Bao J. Xia Y. Zhang J. Zhang L. (2013). The correlation between apparent diffusion coefficient and tumor cellularity in patients: a meta-analysis. *PLoS One* 8:e79008. 10.1371/journal.pone.0079008 24244402 PMC3823989

[B10] Chen L.-P. Jiang X.-Q. Zhao Z. Wang X.-H. (2018). Apparent diffusion coeffcient value for prediction of hemorrhagic transformation in acute ischemic infarction. *Int. J. Clin. Exp. Med.* 11 109–117.

[B11] Collewet G. Strzelecki M. Mariette F. (2004). Influence of MRI acquisition protocols and image intensity normalization methods on texture classification. *Magn. Reson. Imaging* 22 81–91. 10.1016/j.mri.2003.09.001 14972397

[B12] Conte G. M. Castellano A. Altabella L. Iadanza A. Cadioli M. Falini A. (2017). Reproducibility of dynamic contrast-enhanced MRI and dynamic susceptibility contrast MRI in the study of brain gliomas: a comparison of data obtained using different commercial software. *Radiol. Med.* 122 294–302. 10.1007/s11547-016-0720-8 28070841

[B13] Cui Y. Tha K. K. Terasaka S. Yamaguchi S. Wang J. Kudo K. (2016). Prognostic imaging biomarkers in glioblastoma: development and independent validation on the basis of multiregion and quantitative analysis of MR images. *Radiology* 278 546–553. 10.1148/radiol.2015150358 26348233 PMC4734164

[B14] Del Mar Alvarez-Torres M. Juan-Albarracin J. Fuster-Garcia E. Bellvis-Bataller F. Lorente D. Reynes G. (2020). Robust association between vascular habitats and patient prognosis in glioblastoma: an international multicenter study. *J. Magn. Reson. Imaging* 51 1478–1486. 10.1002/jmri.26958 31654541

[B15] Dextraze K. Saha A. Kim D. Narang S. Lehrer M. Rao A. (2017). Spatial habitats from multiparametric MR imaging are associated with signaling pathway activities and survival in glioblastoma. *Oncotarget* 8 112992–113001. 10.18632/oncotarget.22947 29348883 PMC5762568

[B16] Dubinski D. Won S. Y. Rauch M. Behmanesh B. Ngassam L. D. C. Baumgarten P. (2021). Association of isocitrate dehydrogenase (IDH) status with edema to tumor ratio and its correlation with immune infiltration in glioblastoma. *Front. Immunol.* 12:627650. 10.3389/fimmu.2021.627650 33868245 PMC8044904

[B17] Ellingson B. M. Malkin M. G. Rand S. D. Connelly J. M. Quinsey C. LaViolette P. S. (2010). Validation of functional diffusion maps (fDMs) as a biomarker for human glioma cellularity. *J. Magn. Reson. Imaging* 31 538–548. 10.1002/jmri.22068 20187195 PMC2903058

[B18] Englander Z. K. Horenstein C. I. Bowden S. G. Chow D. S. Otten M. L. Lignelli A. (2018). Extent of bold vascular dysregulation is greater in diffuse gliomas without isocitrate dehydrogenase 1 R132H mutation. *Radiology* 287 965–972. 10.1148/radiol.2017170790 29369751

[B19] Fathi Kazerooni A. Nabil M. Zeinali Zadeh M. Firouznia K. Azmoudeh-Ardalan F. Frangi A. F. (2018). Characterization of active and infiltrative tumorous subregions from normal tissue in brain gliomas using multiparametric MRI. *J. Magn. Reson. Imaging* 48 938–950. 10.1002/jmri.25963 29412496 PMC6081259

[B20] Fedorov A. Beichel R. Kalpathy-Cramer J. Finet J. Fillion-Robin J. C. Pujol S. (2012). 3D slicer as an image computing platform for the quantitative imaging network. *Magn. Reson. Imaging* 30 1323–1341. 10.1016/j.mri.2012.05.001 22770690 PMC3466397

[B21] Fuster-Garcia E. Juan-Albarracin J. Garcia-Ferrando G. A. Marti-Bonmati L. Aparici-Robles F. Garcia-Gomez J. M. (2018). Improving the estimation of prognosis for glioblastoma patients by MR based hemodynamic tissue signatures. *NMR Biomed* 31 e4006. 10.1002/nbm.4006 30239058

[B22] Gatenby R. A. Grove O. Gillies R. J. (2013). Quantitative imaging in cancer evolution and ecology. *Radiology* 269 8–15. 10.1148/radiol.13122697 24062559 PMC3781355

[B23] Gates E. D. H. Lin J. S. Weinberg J. S. Hamilton J. Prabhu S. S. Hazle J. D. (2019). Guiding the first biopsy in glioma patients using estimated Ki-67 maps derived from MRI: conventional versus advanced imaging. *Neuro Oncol.* 21 527–536. 10.1093/neuonc/noz004 30657997 PMC6422438

[B24] Halmos G. B. Bruine de Bruin L. Langendijk J. A. van der Laan B. F. Pruim J. Steenbakkers R. J. (2014). Head and neck tumor hypoxia imaging by 18F-fluoroazomycin-arabinoside (18F-FAZA)-PET: a review. *Clin. Nucl. Med.* 39 44–48. 24152663 10.1097/RLU.0000000000000286

[B25] Hardee M. E. Zagzag D. (2012). Mechanisms of glioma-associated neovascularization. *Am. J. Pathol.* 181 1126–1141. 10.1016/j.ajpath.2012.06.030 22858156 PMC3463636

[B26] Holash J. Maisonpierre P. C. Compton D. Boland P. Alexander C. R. Zagzag D. (1999). Vessel cooption, regression, and growth in tumors mediated by angiopoietins and VEGF. *Science* 284 1994–1998. 10.1126/science.284.5422.1994 10373119

[B27] Hu L. S. Ning S. Eschbacher J. M. Gaw N. Dueck A. C. Smith K. A. (2015). Multi-parametric MRI and texture analysis to visualize spatial histologic heterogeneity and tumor extent in glioblastoma. *PLoS One* 10:e0141506. 10.1371/journal.pone.0141506 26599106 PMC4658019

[B28] Huang W. J. Chen W. W. Zhang X. (2016). Glioblastoma multiforme: effect of hypoxia and hypoxia inducible factors on therapeutic approaches. *Oncol. Lett.* 12 2283–2288. 10.3892/ol.2016.4952 27698790 PMC5038353

[B29] Ismail M. Hill V. Statsevych V. Huang R. Prasanna P. Correa R. (2018). Shape features of the lesion habitat to differentiate brain tumor progression from pseudoprogression on routine multiparametric MRI: a multisite study. *AJNR Am. J. Neuroradiol.* 39 2187–2193. 10.3174/ajnr.A5858 30385468 PMC6529206

[B30] Jackson R. J. Fuller G. N. Abi-Said D. Lang F. F. Gokaslan Z. L. Shi W. M. (2001). Limitations of stereotactic biopsy in the initial management of gliomas. *Neuro Oncol.* 3 193–200. 10.1093/neuonc/3.3.193 11465400 PMC1920616

[B31] John F. Bosnyak E. Robinette N. L. Amit-Yousif A. J. Barger G. R. Shah K. D. (2019). Multimodal imaging-defined subregions in newly diagnosed glioblastoma: impact on overall survival. *Neuro Oncol.* 21 264–273. 10.1093/neuonc/noy169 30346623 PMC6374760

[B32] Juan-Albarracin J. Fuster-Garcia E. Garcia-Ferrando G. A. Garcia-Gomez J. M. (2019). ONCOhabitats: a system for glioblastoma heterogeneity assessment through MRI. *Int. J. Med. Inform.* 128 53–61. 10.1016/j.ijmedinf.2019.05.002 31160012

[B33] Juan-Albarracin J. Fuster-Garcia E. Perez-Girbes A. Aparici-Robles F. Alberich-Bayarri A. Revert-Ventura A. (2018). Glioblastoma: vascular habitats detected at preoperative dynamic susceptibility-weighted contrast-enhanced perfusion MR imaging predict survival. *Radiology* 287 944–954. 10.1148/radiol.2017170845 29357274

[B34] Jun P. Garcia J. Tihan T. McDermott M. W. Cha S. (2006). Perfusion MR imaging of an intracranial collision tumor confirmed by image-guided biopsy. *AJNR Am. J. Neuroradiol.* 27 94–97. 16418364 PMC7976103

[B35] Kang B. K. Na D. G. Ryoo J. W. Byun H. S. Roh H. G. Pyeun Y. S. (2001). Diffusion-weighted MR imaging of intracerebral hemorrhage. *Korean J. Radiol.* 2 183–191. 10.3348/kjr.2001.2.4.183 11754324 PMC2718119

[B36] Kim J. Y. Gatenby R. A. (2017). Quantitative clinical imaging methods for monitoring intratumoral evolution. *Methods Mol. Biol.* 1513 61–81. 10.1007/978-1-4939-6539-7_6 27807831

[B37] Kim M. Park J. E. Kim H. S. Kim N. Park S. Y. Kim Y. H. (2021). Spatiotemporal habitats from multiparametric physiologic MRI distinguish tumor progression from treatment-related change in post-treatment glioblastoma. *Eur. Radiol.* 31 6374–6383. 10.1007/s00330-021-07718-y 33569615

[B38] Komori T. Muragaki Y. Chernov M. F. (2018). Pathology and genetics of gliomas. *Prog. Neurol. Surg.* 31 1–37. 10.1159/000466835 29393190

[B39] Lee J. Narang S. Martinez J. Rao G. Rao A. (2015a). Spatial habitat features derived from multiparametric magnetic resonance imaging data are associated with molecular subtype and 12-month survival status in glioblastoma multiforme. *PLoS One* 10:e0136557. 10.1371/journal.pone.0136557 26368923 PMC4569439

[B40] Lee J. Narang S. Martinez J. J. Rao G. Rao A. (2015b). Associating spatial diversity features of radiologically defined tumor habitats with epidermal growth factor receptor driver status and 12-month survival in glioblastoma: methods and preliminary investigation. *J. Med. Imaging (Bellingham)* 2:041006. 10.1117/1.JMI.2.4.041006 PMC4718420 26835490

[B41] Li C. Yan J. L. Torheim T. McLean M. A. Boonzaier N. R. Zou J. (2019). Low perfusion compartments in glioblastoma quantified by advanced magnetic resonance imaging and correlated with patient survival. *Radiother. Oncol.* 134 17–24. 10.1016/j.radonc.2019.01.008 31005212 PMC6486398

[B42] Li Z. C. Bai H. Sun Q. Li Q. Liu L. Zou Y. (2018a). Multiregional radiomics features from multiparametric MRI for prediction of MGMT methylation status in glioblastoma multiforme: a multicentre study. *Eur. Radiol.* 28 3640–3650. 10.1007/s00330-017-5302-1 29564594

[B43] Li Z. C. Bai H. Sun Q. Zhao Y. Lv Y. Zhou J. (2018b). Multiregional radiomics profiling from multiparametric MRI: identifying an imaging predictor of IDH1 mutation status in glioblastoma. *Cancer Med.* 7 5999–6009. 10.1002/cam4.1863 30426720 PMC6308047

[B44] Louis D. N. Perry A. Reifenberger G. von Deimling A. Figarella-Branger D. Cavenee W. K. (2016). The 2016 world health organization classification of tumors of the central nervous system: a summary. *Acta Neuropathol.* 131 803–820. 10.1007/s00401-016-1545-1 27157931

[B45] Lu Y. Yeung C. Radmanesh A. Wiemann R. Black P. M. Golby A. J. (2015). Comparative effectiveness of frame-based, frameless, and intraoperative magnetic resonance imaging-guided brain biopsy techniques. *World Neurosurg.* 83 261–268. 10.1016/j.wneu.2014.07.043 25088233 PMC4450019

[B46] Maier S. E. Sun Y. Mulkern R. V. (2010). Diffusion imaging of brain tumors. *NMR Biomed.* 23 849–864. 10.1002/nbm.1544 20886568 PMC3000221

[B47] Mapelli P. Picchio M. (2020). 18F-FAZA PET imaging in tumor hypoxia: a focus on high-grade glioma. *Int. J. Biol. Markers* 35(1_suppl) 42–46. 10.1177/1724600820905715 32079461

[B48] Mapelli P. Callea M. Fallanca F. Castellano A. Bailo M. Scifo P. (2021). 18F-FAZA PET/CT in pretreatment assessment of hypoxic status in high-grade glioma: correlation with hypoxia immunohistochemical biomarkers. *Nucl. Med. Commun.* 42 763–771. 10.1097/MNM.0000000000001396 33741855

[B49] Mikkelsen V. E. Stensjoen A. L. Berntsen E. M. Nordrum I. S. Salvesen O. Solheim O. (2018). Histopathologic features in relation to pretreatment tumor growth in patients with glioblastoma. *World Neurosurg.* 109 e50–e58. 10.1016/j.wneu.2017.09.102 28951271

[B50] Miloushev V. Z. Chow D. S. Filippi C. G. (2015). Meta-analysis of diffusion metrics for the prediction of tumor grade in gliomas. *AJNR Am. J. Neuroradiol.* 36 302–308. 10.3174/ajnr.A4097 25190201 PMC7965672

[B51] Muragaki Y. Chernov M. Maruyama T. Ochiai T. Taira T. Kubo O. (2008). Low-grade glioma on stereotactic biopsy: how often is the diagnosis accurate? *Minim. Invasive Neurosurg.* 51 275–279. 10.1055/s-0028-1082322 18855292

[B52] Napel S. Mu W. Jardim-Perassi B. V. Aerts H. J. Gillies R. J. (2018). Quantitative imaging of cancer in the postgenomic era: radio (geno) mics, deep learning, and habitats. *Cancer* 124 4633–4649. 10.1002/cncr.31630 30383900 PMC6482447

[B53] Nguyen T. B. Cron G. O. Bezzina K. Perdrizet K. Torres C. H. Chakraborty S. (2016). Correlation of tumor immunohistochemistry with dynamic contrast-enhanced and DSC-MRI parameters in patients with gliomas. *AJNR Am. J. Neuroradiol.* 37 2217–2223. 10.3174/ajnr.A4908 27585700 PMC7963881

[B54] O’Connor J. P. Rose C. J. Waterton J. C. Carano R. A. Parker G. J. Jackson A. (2015). Imaging intratumor heterogeneity: role in therapy response, resistance, and clinical outcome. *Clin. Cancer Res.* 21 249–257. 10.1158/1078-0432.CCR-14-0990 25421725 PMC4688961

[B55] Ono Y. Chernov M. F. Muragaki Y. Maruyama T. Abe K. Iseki H. (2018). Imaging of Intracranial Gliomas. *Prog. Neurol. Surg.* 30 12–62. 10.1159/000464376 29241169

[B56] Otsu N. (1979). A threshold selection method from gray-level histograms. *IEEE Trans. Syst Man Cybern.* 9 62–66. 10.1109/TSMC.1979.4310076

[B57] Park J. E. Kim H. S. Kim N. Park S. Y. Kim Y. H. Kim J. H. (2021). Spatiotemporal heterogeneity in multiparametric physiologic MRI Is associated with patient outcomes in IDH-wildtype glioblastoma. *Clin. Cancer Res.* 27 237–245. 10.1158/1078-0432.CCR-20-2156 33028594

[B58] Pillai J. J. Zaca D. (2012). Comparison of BOLD cerebrovascular reactivity mapping and DSC MR perfusion imaging for prediction of neurovascular uncoupling potential in brain tumors. *Technol. Cancer Res. Treat.* 11 361–374. 10.7785/tcrt.2012.500284 22376130

[B59] Ponte K. F. Berro D. H. Collet S. Constans J. M. Emery E. Valable S. (2017). In vivo relationship between hypoxia and angiogenesis in human glioblastoma: a multimodal imaging study. *J. Nucl. Med.* 58 1574–1579. 10.2967/jnumed.116.188557 28596159

[B60] Preibisch C. Shi K. Kluge A. Lukas M. Wiestler B. Gottler J. (2017). Characterizing hypoxia in human glioma: a simultaneous multimodal MRI and PET study. *NMR Biomed.* 30 e3775. 10.1002/nbm.3775 28805936

[B61] Reithmeier T. Lopez W. O. Doostkam S. Machein M. R. Pinsker M. O. Trippel M. (2013). Intraindividual comparison of histopathological diagnosis obtained by stereotactic serial biopsy to open surgical resection specimen in patients with intracranial tumours. *Clin. Neurol. Neurosurg.* 115 1955–1960. 10.1016/j.clineuro.2013.05.019 23769864

[B62] Rosinska S. Gavard J. (2021). Tumor vessels fuel the fire in glioblastoma. *Int. J. Mol. Sci.* 22:6514. 10.3390/ijms22126514 34204510 PMC8235363

[B63] Roth A. Buttrick S. S. Cajigas I. Jagid J. R. Ivan M. E. (2018). Accuracy of frame-based and frameless systems for deep brain stimulation: a meta-analysis. *J. Clin. Neurosci.* 57 1–5. 10.1016/j.jocn.2018.08.039 30197058

[B64] Rotkopf L. T. Wiestler B. Preibisch C. Liesche-Starnecker F. Pyka T. Norenberg D. (2020). The wavelet power spectrum of perfusion weighted MRI correlates with tumor vascularity in biopsy-proven glioblastoma samples. *PLoS One* 15:e0228030. 10.1371/journal.pone.0228030 31971966 PMC6977746

[B65] Santarosa C. Castellano A. Conte G. M. Cadioli M. Iadanza A. Terreni M. R. (2016). Dynamic contrast-enhanced and dynamic susceptibility contrast perfusion MR imaging for glioma grading: preliminary comparison of vessel compartment and permeability parameters using hotspot and histogram analysis. *Eur. J. Radiol.* 85 1147–1156. 10.1016/j.ejrad.2016.03.020 27161065

[B66] Sanvito F. Castellano A. Falini A. (2021). Advancements in neuroimaging to unravel biological and molecular features of brain tumors. *Cancers (Basel)* 13:424. 10.3390/cancers13030424 33498680 PMC7865835

[B67] Sauwen N. Acou M. Van Cauter S. Sima D. M. Veraart J. Maes F. (2016). Comparison of unsupervised classification methods for brain tumor segmentation using multi-parametric MRI. *Neuroimage Clin.* 12 753–764. 10.1016/j.nicl.2016.09.021 27812502 PMC5079350

[B68] Savi A. Incerti E. Fallanca F. Bettinardi V. Rossetti F. Monterisi C. (2017). First evaluation of pet-based human biodistribution and dosimetry of (18)F-FAZA, a tracer for imaging tumor hypoxia. *J. Nucl. Med.* 58 1224–1229. 10.2967/jnumed.113.122671 28209906

[B69] Sciortino T. Fernandes B. Conti Nibali M. Gay L. G. Rossi M. Lopci E. (2019). Frameless stereotactic biopsy for precision neurosurgery: diagnostic value, safety, and accuracy. *Acta Neurochir. (Wien)* 161 967–974. 10.1007/s00701-019-03873-w 30895395

[B70] Sottoriva A. Spiteri I. Piccirillo S. G. Touloumis A. Collins V. P. Marioni J. C. (2013). Intratumor heterogeneity in human glioblastoma reflects cancer evolutionary dynamics. *Proc. Natl. Acad. Sci. U.S.A.* 110 4009–4014. 10.1073/pnas.1219747110 23412337 PMC3593922

[B71] Stadlbauer A. Buchfelder M. Doelken M. T. Hammen T. Ganslandt O. (2011). Magnetic resonance spectroscopic imaging for visualization of the infiltration zone of glioma. *Cent. Eur. Neurosurg.* 72 63–69. 10.1055/s-0030-1253410 20635312

[B72] Stadlbauer A. Zimmermann M. Doerfler A. Oberndorfer S. Buchfelder M. Coras R. (2018b). Intratumoral heterogeneity of oxygen metabolism and neovascularization uncovers 2 survival-relevant subgroups of IDH1 wild-type glioblastoma. *Neuro Oncol.* 20 1536–1546. 10.1093/neuonc/noy066 29718366 PMC6176796

[B73] Stadlbauer A. Mouridsen K. Doerfler A. Bo Hansen M. Oberndorfer S. Zimmermann M. (2018a). Recurrence of glioblastoma is associated with elevated microvascular transit time heterogeneity and increased hypoxia. *J. Cereb. Blood Flow Metab.* 38 422–432. 10.1177/0271678X17694905 28273720 PMC5851132

[B74] Stringfield O. Arrington J. A. Johnston S. K. Rognin N. G. Peeri N. C. Balagurunathan Y. (2019). Multiparameter MRI predictors of long-term survival in glioblastoma multiforme. *Tomography* 5 135–144. 10.18383/j.tom.2018.00052 30854451 PMC6403044

[B75] Verma R. Correa R. Hill V. B. Statsevych V. Bera K. Beig N. (2020). Tumor habitat-derived radiomic features at pretreatment mri that are prognostic for progression-free survival in glioblastoma are associated with key morphologic attributes at histopathologic examination: a feasibility study. *Radiol. Artif. Intell.* 2:e190168. 10.1148/ryai.2020190168 33330847 PMC7706886

[B76] Wang Q. Li Q. Mi R. Ye H. Zhang H. Chen B. (2019). Radiomics nomogram building from multiparametric MRI to predict grade in patients with glioma: a cohort study. *J. Magn. Reson. Imaging* 49 825–833. 10.1002/jmri.26265 30260592

[B77] Wei J. Yang G. Hao X. Gu D. Tan Y. Wang X. (2019). A multi-sequence and habitat-based MRI radiomics signature for preoperative prediction of MGMT promoter methylation in astrocytomas with prognostic implication. *Eur. Radiol.* 29 877–888. 10.1007/s00330-018-5575-z 30039219 PMC6302873

[B78] Winfield J. M. Payne G. S. Weller A. deSouza N. M. (2016). DCE-MRI, DW-MRI, and MRS in cancer: challenges and advantages of implementing qualitative and quantitative multi-parametric imaging in the clinic. *Top. Magn. Reson. Imaging* 25 245–254. 10.1097/RMR.0000000000000103 27748710 PMC5081190

[B79] Wu H. Tong H. Du X. Guo H. Ma Q. Zhang Y. (2020). Vascular habitat analysis based on dynamic susceptibility contrast perfusion MRI predicts IDH mutation status and prognosis in high-grade gliomas. *Eur. Radiol.* 30 3254–3265. 10.1007/s00330-020-06702-2 32078014

[B80] Yip C. Weeks A. Shaw K. Siddique M. Chang F. Landau D. B. (2017). Magnetic resonance imaging (MRI) of intratumoral voxel heterogeneity as a potential response biomarker: assessment in a HER2+ esophageal adenocarcinoma xenograft following trastuzumab and/or cisplatin therapy. *Transl. Oncol.* 10 459–467. 10.1016/j.tranon.2017.03.006 28456115 PMC5408154

[B81] Zhang J. Liu H. Tong H. Wang S. Yang Y. Liu G. (2017). Clinical applications of contrast-enhanced perfusion mri techniques in gliomas: recent advances and current challenges. *Contrast Media Mol. Imaging* 2017:7064120. 10.1155/2017/7064120 29097933 PMC5612612

[B82] Zhang X. Lu H. Tian Q. Feng N. Yin L. Xu X. (2019). A radiomics nomogram based on multiparametric MRI might stratify glioblastoma patients according to survival. *Eur. Radiol.* 29 5528–5538. 10.1007/s00330-019-06069-z 30847586

[B83] Zhou M. Chaudhury B. Hall L. O. Goldgof D. B. Gillies R. J. Gatenby R. A. (2017). Identifying spatial imaging biomarkers of glioblastoma multiforme for survival group prediction. *J. Magn. Reson. Imaging* 46 115–123. 10.1002/jmri.25497 27678245

[B84] Zhou M. Hall L. Goldgof D. Russo R. Balagurunathan Y. Gillies R. (2014). Radiologically defined ecological dynamics and clinical outcomes in glioblastoma multiforme: preliminary results. *Transl. Oncol.* 7 5–13. 10.1593/tlo.13730 24772202 PMC3998688

