# Supplementary material for: Decoding the Heterogeneity of Malignant Gliomas by PET and MRI for Spatial Habitat Analysis of Hypoxia, Perfusion, and Diffusion Imaging: A Preliminary Study
Source: Front Neurosci. 2022 Jul 13;16:885291. doi: 10.3389/fnins.2022.885291 (PMC9326318; doi:10.3389/fnins.2022.885291)
Supplement: Supplementary file 1 [file Table_1.pdf]

Supplementary Material

Supplementary Table 1 - Characteristics of the sequences acquired on the 1.5T MR scanner.

	Scanning sequence and options	TR (ms)	TE (ms)	FOV (mm)	Acquisition matrix (mm)	Slice thickness (mm)	Reconstructed voxel (mm)	Acquisition time (seconds)
3D T1 CE	Axial 3D spoiled gradient echo T1 weighted	25	5	256	256x256	1	1x1x1	512
3D FLAIR	Axial 3D FLAIR (TI = 1700)	4800	320	240	240x240	1	1x1x1	240
DTI	GE-EPI, b-values of 0 and 1000 s/mm^2, 15 directions	3733	99	224	92x90	2.5	1.75x1.75x2.5	694
DCE	Axial DCE 3D spoiled gradient echo T1 weighted, 70 dynamics	4	1.9	230	96x96	5	2.05x2.05x2.5	466
DSC	Axial DSC FFE-EPI T2* weighted, 80 dynamics	1650	40	230	76x61	5	2.05x2.05x5	137

The scanner was a Philips Achieva (Philips Healthcare, Best, Netherlands). GE = gradient eco, EPI = Echo Planar Imaging, IR = inversion recovery, SE = Spin Echo, CE = contrast-enhanced, TI = Time of inversion.

Supplementary Table 2 - Characteristics of the sequences acquired on the 3T MR scanner.

	Scanning sequence and options	TR (ms)	TE (ms)	FOV (mm)	Acquisition matrix (mm)	Slice thickness (mm)	Reconstructed voxel (mm)	Acquisition time (seconds)
3D T1 CE	Axial 3D spoiled gradient echo T1 weighted	11	5	256	284x190	0.9	0.8x0.8x0.9	321
3D FLAIR	Axial 3D FLAIR (TI = 1700)	9000	300	230	204x204	1.2	0.7x0.7x0.7	486
DTI	GE-EPI, b-values of 0 and 1000 s/mm^2, 32 directions	3200	80	240	96x94	2.5	0.94x0.94x2.5	215
DCE	Axial DCE 3D spoiled gradient echo T1 weighted, 70 dynamics	4	1.8	230	96x96	5	2.05x2.05x2.5	382
DSC	Axial DSC FFE-EPI T2* weighted, 80 dynamics	1500	40	230	96x78	5	2.05x2.05x5	124

The scanner was an Ingenia CX (Philips Healthcare, Best, Netherlands). GE = gradient eco, EPI = Echo Planar Imaging, IR = inversion recovery, SE = Spin Echo, CE = contrast-enhanced, TI = Inversion Time
