# Supplementary material for: Decoding the Heterogeneity of Malignant Gliomas by PET and MRI for Spatial Habitat Analysis of Hypoxia, Perfusion, and Diffusion Imaging: A Preliminary Study
Source: Front Neurosci. 2022 Jul 13;16:885291. doi: 10.3389/fnins.2022.885291 (PMC9326318; doi:10.3389/fnins.2022.885291)
Supplement: Supplementary file 2 [file Image_1.pdf]

Supplementary Material

Supplementary Figure 1 - OTSU algorithm and cluster intersection.

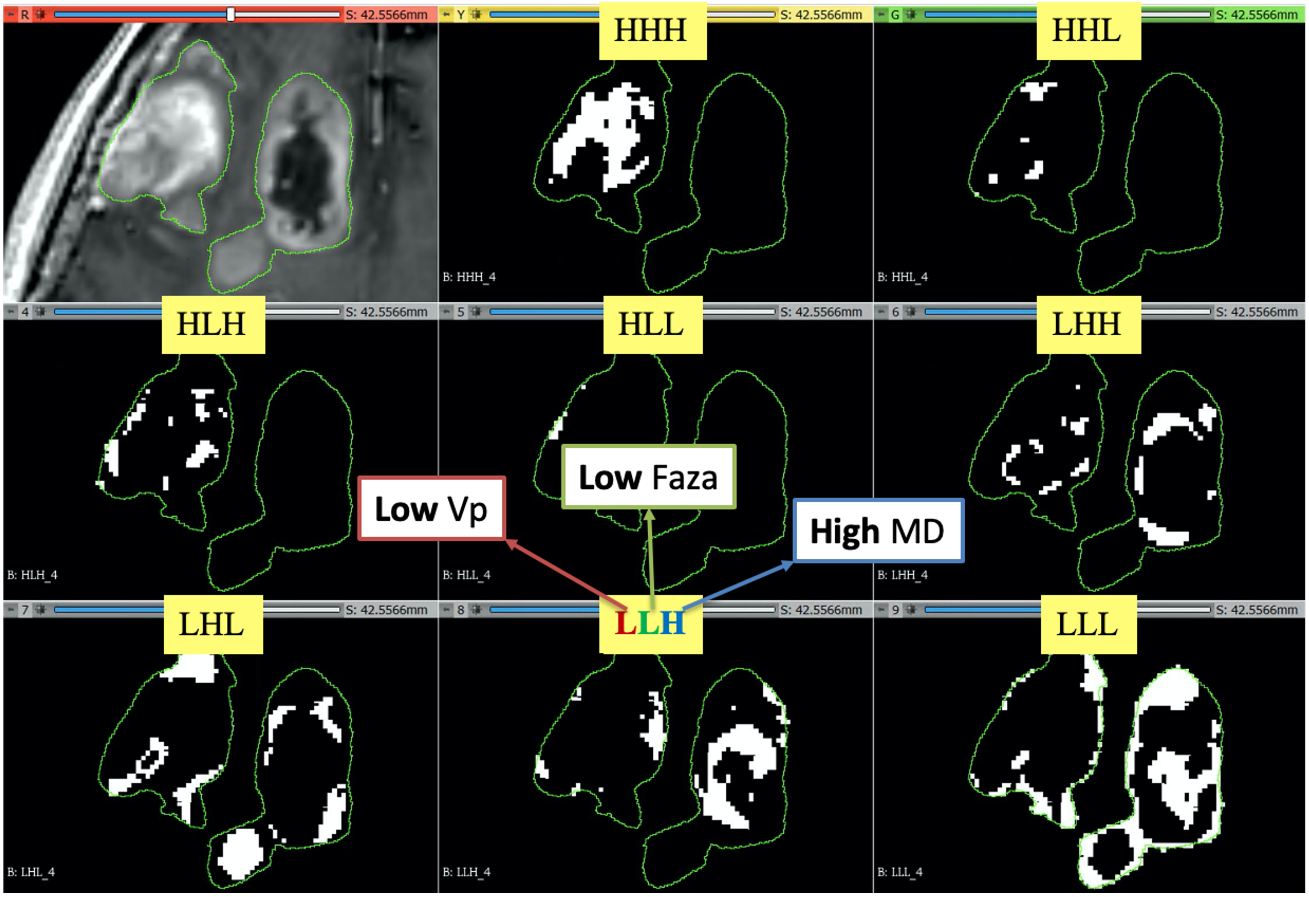

The OTSU algorithm was adopted to identify the best cut-off able to divide voxels in two cluster of intensity while preserving the highest possible amount of information. Each tumor-masked parametric map then underwent an automatic OTSU binary clustering in 2 disjointed regions by grouping together voxels with HIGH and LOW intensity values. The 8 combinations (cluster intersection) were then generated by combining all possible high (H) and low (L) region of each map (e.g., Vp + FAZA + MD) obtained from the OTSU algorithm.
